# Biomineralization To Prevent Microbially Induced Corrosion on Concrete for Sustainable Marine Infrastructure

**DOI:** 10.1021/acs.est.3c04680

**Published:** 2023-12-05

**Authors:** Xiaohao Sun, Onyx W. H. Wai, Jiawen Xie, Xiangdong Li

**Affiliations:** † Department of Civil and Environmental Engineering, The Hong Kong Polytechnic University, Hung Hom, Kowloon, Hong Kong SAR, China; ‡ Research Institute for Sustainable Urban Development, The Hong Kong Polytechnic University, Hung Hom, Kowloon, Hong Kong, SAR, China

**Keywords:** sustainable marine concrete, MIC, biomineralization, corrosion inhibition, SRB community

## Abstract

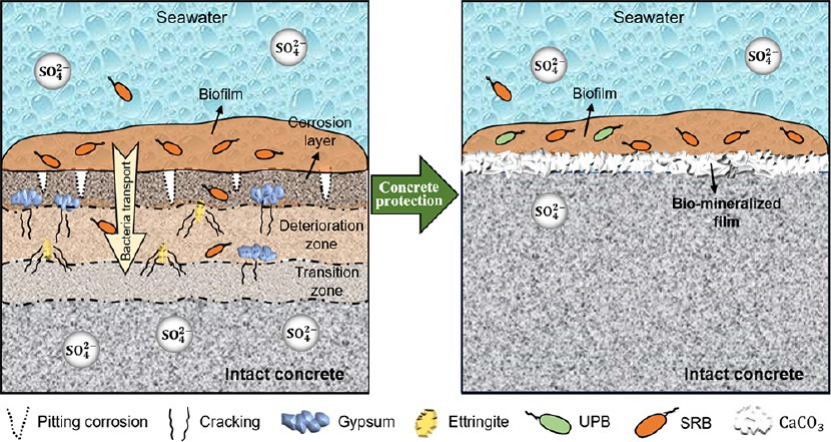

Microbially induced corrosion (MIC) on concrete represents a serious issue impairing the lifespan of coastal/marine infrastructure. However, currently developed concrete corrosion protection strategies have limitations in wide applications. Here, a biomineralization method was proposed to form a biomineralized film on concrete surfaces for corrosion inhibition. Laboratory seawater corrosion experiments were conducted under different conditions [e.g., chemical corrosion (CC), MIC, and biomineralization for corrosion inhibition]. A combination of chemical and mechanical property measurements of concrete (e.g., sulfate concentrations, permeability, mass, and strength) and a genotypic-based investigation of formed concrete biofilms was conducted to evaluate the effectiveness of the biomineralization approach on corrosion inhibition. The results show that MIC resulted in much higher corrosion rates than CC. However, the biomineralization treatment effectively inhibited corrosion because the biomineralized film decreased the total and relative abundance of sulfate-reducing bacteria (SRB) and acted as a protective layer to control the diffusion of sulfate and isolate the concrete from the corrosive SRB communities, which helps extend the lifespan of concrete structures. Moreover, this technique had no negative impact on the native marine microbial communities. Our study contributes to the potential application of biomineralization for corrosion inhibition to achieve long-term sustainability for major marine concrete structures.

## Abstract 2

A novel biomineralization strategy is proposed to isolate marine concrete from microbially induced corrosion (MIC) to achieve sustainable coastal structures.

## 1 Introduction

Oceans are extremely rich in resources, and their development and utilization are essential to society, especially for coastal cities, which rely heavily on their coastal and marine infrastructure for social-economic development. In marine environments, microbially induced corrosion (MIC) is a common phenomenon leading to a global economic loss of ∼US$800 billion annually.^1^ Concrete has been used extensively as a construction material for coastal, marine, and offshore engineering developments. However, MIC on concrete usually occurs in harsh environments with the presence of corrosive microorganisms, such as sewage structures, wastewater treatment plants, and marine infrastructure.^2−5^ The process of MIC on concrete can be divided into several stages:^6^ (1) sulfate (SO_4_^2–^) is converted to sulfide (H_2_S) through the biological activities of sulfate-reducing bacteria (SRB) residing in biofilms. (2) H_2_S is then converted to sulfuric acid (H_2_SO_4_) by sulfur-oxidizing bacteria (SOB). (3) Sulfuric acid reacts with the concrete matrix to form gypsum and ettringite, leading to expansion stress and matrix fractures. It is well-known that seawater contains high concentrations of aggressive ions, and the related chemical corrosion is thought to be a frequent problem.^7^ However, different from the concrete-based wastewater networks,^8,9^ insufficient attention has been paid to the effect of MIC on marine concrete degradation, and the importance of protection from MIC is not widely recognized. As corrosive microorganisms can accelerate the corrosion of concrete,^10^ the presence of MIC easily results in concrete cracking, which decreases the lifespan of concrete structures^11^ and causes considerable economic loss.^12^ Thus, this is one of the major obstacles to the long-term use of marine concrete structures that must be solved to achieve sustainable coastal cities.

The annual cost to rehabilitate/restore MIC concrete structures is estimated at £85 million in the U.K. and over €450 million in Germany.^9^ To reduce the economic investment and energy consumption involved in restoration, several methods have been developed to control corrosion [e.g., biocides, adding supplementary cementitious materials (SCMs), and new types of concrete]. However, high dosages of biocides are required in the field to treat biofilms, and repeated treatments may cause these species to become more tolerant/resistant to the biocides.^13^ Intermittent dosages of free nitrous acid have been demonstrated to be effective at mitigating metal corrosion in a simulated water injection system;^14^ however, the feasibility of this approach is yet to be tested on marine concrete corrosion inhibition. The addition of SCMs not only improves the mechanical performance of concrete but also reduces the ingress of aggressive ions, thereby mitigating corrosion.^15^ Several new types of concrete [e.g., seawater sea sand concrete (SSC), calcium aluminate concrete (CAC), and alkali-activated concrete (AAC)] also exhibited superior resistance performance under the ingress of sulfate ions.^10,16,17^ However, the performance of the addition of SCMs and new types of concrete is yet to be proven in MIC environments. Therefore, there is still an increasing demand for new “green” and effective alternatives to inhibit the corrosion of marine concrete.

Biofilms can have both a detrimental and beneficial effect on corrosion resistance.^18^ Protective biofilms can provide a barrier to inhibit corrosion, and their formation is typically considered to be the major anticorrosion mechanism.^19^ SRB communities have been confirmed as the dominant species contributing to marine corrosion^20^ and can be used to evaluate the risk of MIC for hydraulic concrete structures.^21^ Controlling SRB communities in the early stage by forming protective biofilms is an effective and efficient way to inhibit corrosion. Some bacteria in marine ecosystems can produce precipitates that combine with other substances to form an organic–inorganic hybrid biomineralized film on the material surface for long-term protection.^22^ For example, a hybrid film (composed of calcite and bacterial extracellular polymeric substances) was designed to protect steel from corrosion in seawater.^23^ Recently, biomineralization or microbially induced carbonate precipitation has attracted extensive attention from researchers in the fields of civil and environmental engineering,^24−26^ which has provided novel insights into the protection of marine concrete. Urea hydrolysis, the commonly used pathway to biomineralization, refers to the binding of metal ions with acid radical ions to form minerals (e.g., calcium carbonate, CaCO_3_).^27,28^ Attention has been paid to using the biomineralization technique to seal concrete cracks,^29,30^ improve the durability of concrete,^25,31^ and stabilize sand foreshore slopes^32^ in marine engineering projects. There are many studies on biomineralization for marine concrete crack repair;^33^ however, few studies have focused on applying the biomineralization approach to inhibit marine concrete corrosion.

Based on the reported corrosion control work on metal materials,^23^ we hypothesize that biomineralization would also be able to inhibit the corrosion of marine concrete structures. However, the inhibition effect of biomineralization on concrete corrosion cannot be inferred from previous metal work due to different corrosion mechanisms. In this study, the biomineralization method was proposed for protecting marine concrete. A laboratory seawater corrosion experiment was conducted to test this hypothesis. Existing test methods employed to evaluate corrosion (e.g., mass loss, surface pH, and compressive strength reduction) mainly focus on the properties of concrete and may not be appropriate to address the interaction between bacteria and concrete. Other methods, such as measuring the concentration of sulfate, are indirect representations of the biological sulfate cycle during corrosion and also fail to represent actual bacterial activity. Molecular techniques can provide detailed information about the composition of microbial communities. Thus, a combination of measurements of the mechanical properties of concrete and an analysis of the microbial community of biofilms was conducted to gain a better understanding of the development of MIC with the aim of evaluating the effectiveness of using biomineralization techniques to inhibit the corrosion of marine concrete. Moreover, the microscopic characteristics of concrete biofilms were carefully examined by using scanning electron microscopy with energy-dispersive X-ray spectroscopy (SEM-EDX). The results obtained from this study will contribute to the development of new techniques for inhibiting corrosion to achieve long-term sustainable marine concrete structures.

## 2 Materials and Methods

### 2.1 Preparing Concrete Specimens

A major challenge for marine infrastructure is the shortage of fresh water and river sand for making concrete.^34^ Apart from the negative environmental effects of consuming great amounts of fresh water and river sand, their transportation can be both expensive and environmentally detrimental. Using SSC is beneficial for the sustainability of coastal cities;^35^ thus, the SSC specimens were prepared in this study by mixing Portland cement, pulverized fuel ash, aggregates, sea sand, seawater, and superplasticizers (the mix proportions are shown in Table S1). The resulting paste was placed in a custom-made frame (10 × 10 × 10 cm) and then cured in a standard curing room at 20 ± 2 °C and 95% relative humidity for 28 days.

### 2.2 Laboratory Seawater Corrosion Experiment

Before conducting the corrosion experiment, urease-producing bacteria (UPB) in seawater were enriched using different enrichment media (Table S2), and their ureolytic capacities were compared (Figure S1) in order to obtain the optimum medium (20 g/L yeast extract, 100 mM urea, and 50 mM ammonium chloride) for biomineralization (the details have been presented in Section S2). In this experiment, three groups of SSC specimens were prepared with biomineralization for concrete corrosion inhibition (BCCI) corresponding to different concentrations of bacterial cells (about 1 × 10^6^ CFU/mL, 5 × 10^6^ CFU/mL, and 1 × 10^7^ CFU/mL) to compare the inhibition effects (Table S3), where each surface was coated with a mixture of 12 mL of bacterial suspension and 12 mL of 1.0 M urea-calcium chloride solution. In addition, a group of specimens was prepared without any treatment to study the corrosion with naturally attached biofilms (F-0 and T-0). To study the effect of chemical corrosion (CC), a group of specimens was prepared (CF-0 and CT-0), where ethanol was used to remove the surface microorganisms every week. Moreover, three concrete specimens without corrosion were prepared to obtain their original strength. Because of daily tidal variations, concrete structures along the coastline may be subject to submerged and tidal conditions. In addition to different treatments, the two types of inoculation conditions, namely, submerged and tidal, were used in this study. For submerged inoculation, half of the concrete specimens were immersed in seawater for the whole day. For tidal inoculation, the other half was immersed in seawater for 16 h and removed from seawater for 8 h every day to mimic daily variations in the tides.

**Figure 1:**
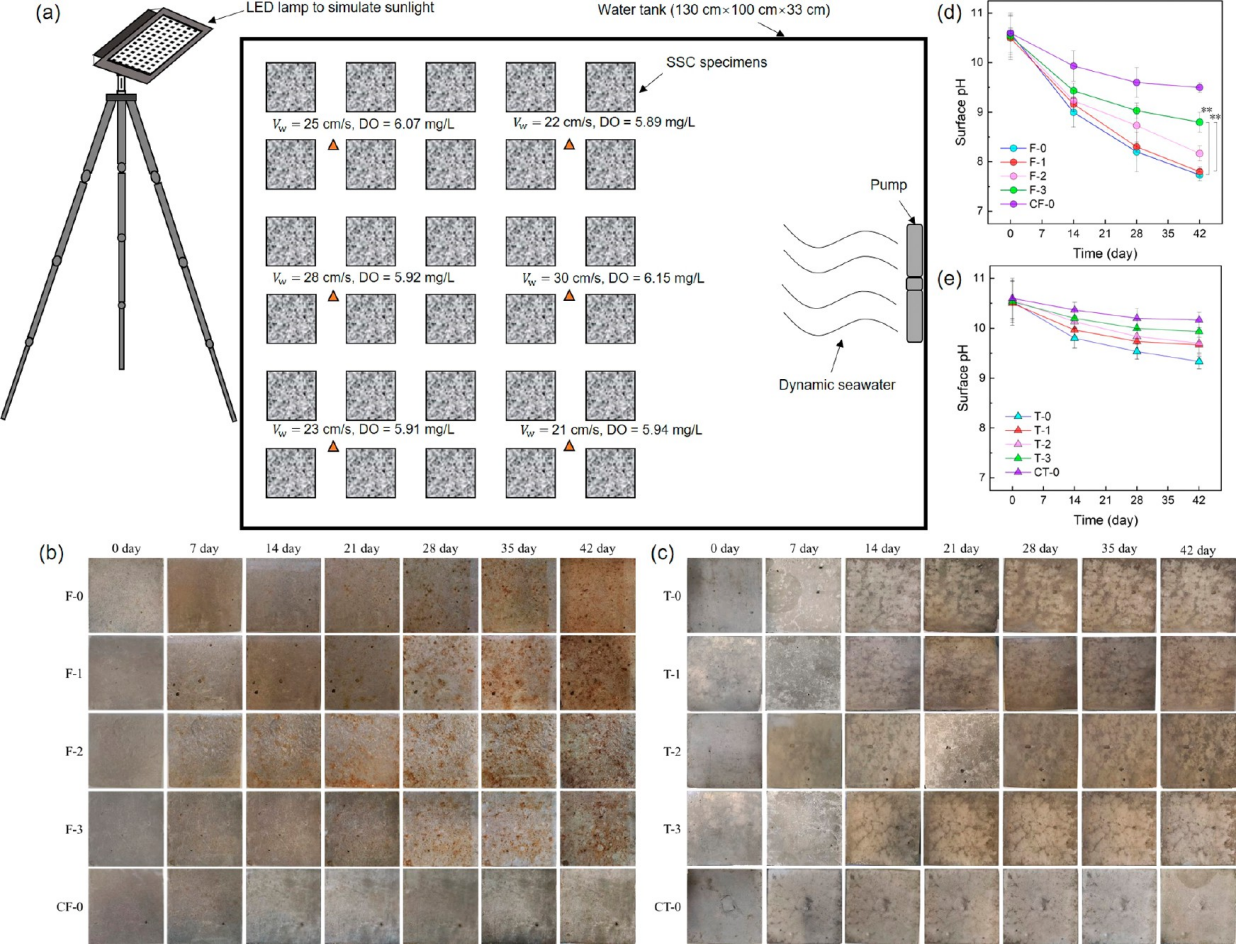
Characteristics of the development of concrete corrosion in a laboratory seawater corrosion experiment. Panel (a) is a schematic diagram of the seawater corrosion experiment. Panels (b) and (c) are the evolution during the experiment for the concrete specimens with submerged inoculation and the concrete specimens with tidal inoculation. Panels (d) and (e) show the evolution of surface pH during the corrosion for the submerged concrete specimens and the tidal concrete specimens, respectively. The error bar represents the standard deviations (** indicates a highly significant difference, *p* < 0.01 and * indicates a significant difference, *p* < 0.05). Note that the SSC in panel (a) is seawater sea sand concrete; Vw and DO refer to the current flow velocity and dissolved oxygen, respectively. The Vw and DO data in this figure were obtained before the corrosion experiment.

After 24 h, all of the concrete specimens were placed in a water tank (Figure 1a). The experiment was conducted at room temperature (18–22 °C) and lasted for 42 days (from 12 January 2022 to 22 February 2022). The main focus was on the bacteria in biofilms because according to the literature, the bacterial communities in concrete biofilms tend to be stable after 42 days.^36^ An LED lamp was used to simulate sunlight.^37^ Real seawater used for the experiment was sampled from the Tsim Sha Tsui Pier in Victoria Harbour. During the experiment, half of the seawater in the water tank was changed every 7 days.^23^ The physicochemical parameters of the seawater sampled from the Tsim Sha Tsui Pier and the seawater in the water tank were measured for comparison (Section S4). The flow velocity of the seawater in the water tank was set at about 20–35 cm/s based on marine data in the dry season, during which the overland runoff discharging into the coastal area is at a minimum^38−40^ (details of the corrosion experimental process are presented in Section S3).

### 2.3 Visual Inspection, Surface pH, Surface Sulfate Concentrations, and CaCO_3_ Contents

During the experiment, photographs were taken every week to observe different patterns of corrosion on the surface of the concrete. A flat surface pH electrode (Extech PH150-C concrete pH kit, Extech Instruments, USA) was used to study the evolution of the pH on the concrete surface during the corrosion process. The concrete specimens were taken out of the tank for surface pH measurement. Four measurement spots on the surface of the concrete were randomly selected to determine the average value. Afterward, specimens were put back in the tank for further exposure.

After the corrosion, the exposed surface of the concrete was washed by using a high-pressure washer with 4000 mL of deionized water to remove biofilms that had formed with surface corrosion products. The soluble sulfate in the wash-off water was analyzed by ion chromatography, and the surface sulfate concentration was calculated.^41^ Moreover, the CaCO_3_ content in the concrete biofilms that had formed was measured using the acid pickling method^42^ (for details of the measurements of surface sulfate concentrations and CaCO_3_ contents, please refer to Section S5).

### 2.4 Mechanical Property Measurements of Concrete Specimens

Before the experiment, the concrete specimens were weighed as the initial mass. After corrosion, the concrete specimens were weighed again to obtain mass loss. In addition to mass loss, compressive strength reduction was also obtained by conducting compressive strength tests as a valuable indicator to study corrosion rates.^43^ Moreover, the apparent volume of permeable voids (AVPV), an indicator of permeability, was obtained following the method described in the literature.^44^

According to Fick’s second law, the sulfate concentrations in concrete are related to corrosion depth and exposure time due to ion diffusion.^45^ The transport of bacteria in pores or cracks affected the concentration of ions in the concrete,^26^ resulting in different corrosion depths and diffusion coefficients. The concrete specimens were ground into powders at different depth intervals. The sulfate concentrations at different depths were obtained using the barium sulfate gravimetric method^46^ (details of the measurements of mass loss, AVPV, strength reduction, and sulfate concentrations in concrete are presented in Section S6).

### 2.5 Analysis of Microbial Communities and Characterizations of the Morphologies of Biofilms

To study the composition and succession of microbial communities, biofilms on the concrete surface were sampled by using a sterilized scalpel every 14 days. The biofilms were carefully washed with Milli-Q water, placed into 2 mL DNA-free aseptic centrifuge tubes, and stored at −20 °C. The biofilm samples were dried under a N_2_ environment. DNA was extracted from the biofilm samples according to the manual of the FastDNA Spin Kit for Soil (MP Biomedicals)^21,47^ and then stored at −80 °C before downstream experiments were conducted. The 16S rRNA gene and β-subunit of the dissimilatory sulfite reductase (*dsrB*) gene were quantified on a StepOnePlus Real-Time PCR System (Applied Biosystems) to assess the total bacterial loading and SRB abundance, respectively^48^ (for details of the qPCR process, please refer to Section S7).

Subsequently, around 25–100 ng of undiluted DNA from each sample was submitted for metagenomics sequencing on a MGISEQ-2000 platform (MGI Tech, China)^49^ with a PE100 strategy. Clean data from the biofilm samples were obtained after sequencing adaptors were trimmed and low-quality reads were filtered out using fastp (v0.21.0 with default parameters).^50^ A taxonomy classification was conducted in Kraken 2 (v2.0.8-beta)^51^ and Bracken (v2.5.0)^52^ using the standard Kraken 2 database and default parameters to compare the bacterial communities at the species level in different concrete biofilms. Furthermore, the Silva Kraken 2 database was used to investigate the proportion of other nonbacteria (e.g., Eukaryota) and their influences on corrosion. The α diversity (Shannon index) was calculated using the “diversity alpha-group-significance” method. A principal coordinate analysis (PCoA) plot of biofilm samples collected on the 14th, 28th, and 42nd day was generated using “diversity beta-group-significance” and “emperor plot” methods based on the unweighted UniFrac distance metrics.^53^ In addition, to investigate changes in the community of sulfur-utilizing bacteria during the corrosion process, the SRB and SOB profiles in different concrete biofilms were screened out from the total bacterial community profile according to a list of commonly studied SRB and SOR from the literature (see Table S6 in Section S8 and Table S7 in Section S9).

The microscopic characteristics and mass percentages of different elements of the concrete biofilms were observed using SEM-EDX (TESCAN VEGA3, TESCAN, Czech Republic).^54,55^ X-ray Diffraction (XRD) (Rigaku SmartLab 9 kW-Advance) was used to identify the corrosion products.^56^

## 3 Results and Discussion

### 3.1 Visual Inspection and Surface pH

The laboratory seawater corrosion experiment was conducted to evaluate the inhibition performance of the proposed biomineralization approach. The results are summarized in Table S8 in Section S10. Figure 1b,c shows that all concrete specimens gradually developed corrosion, and some yellowish corrosion products with an attached biofilm were observed during the 42 day period of the experiment. During the initial 14 days, the biofilm formed in the submerged group, covering a larger area of the surface of the concrete. The corroded layer provided an excellent medium for microorganisms to grow, which consequently accelerated the corrosion process.^57^ Less attached biofilm was observed in the tidal groups, with the least biofilm observed on the CC specimens.

During the corrosion, some physicochemical parameters of the seawater in the tank changed in comparison with those of the field sampled seawater (Tables S4 and S5 in Section S4). The changes in physicochemical parameters (Figure S2) and genotypic data (Figure S4) suggested that a transition occurred from a situation where the major influence on the concrete came from bacteria to the condition where the influence came from a combination of both bacteria and phytoplankton (details can be seen in Section S11). Surface pH is a good indicator of the development of corrosion due to continuous acidification by MIC and subsequent neutralization of cementitious compounds in the concrete.^44^ After only 42 days of exposure, the decrease in the range of pH reached about 3.0 for concrete specimens with MIC (F-0) (Figure 1d), indicating a serious condition of corrosion,^41^ which was significantly larger than that for specimens with CC (CF-0, *p* < 0.01). The BCCI samples had smaller decreasing ranges of pH than F-0. The pH values varied with the UPB concentrations, generally following the order of F-3 > F-2 > F-1. Concerning the tidal groups, their ranges of decrease were much smaller than those of the submerged groups (Figure 1e). Similarly, T-3 with the highest UPB concentration had the smallest range of decreases of surface pH.

### 3.2 Sulfate Concentrations, Apparent Volume of Permeable Voids, and Corrosion Rates

**Figure 2:**
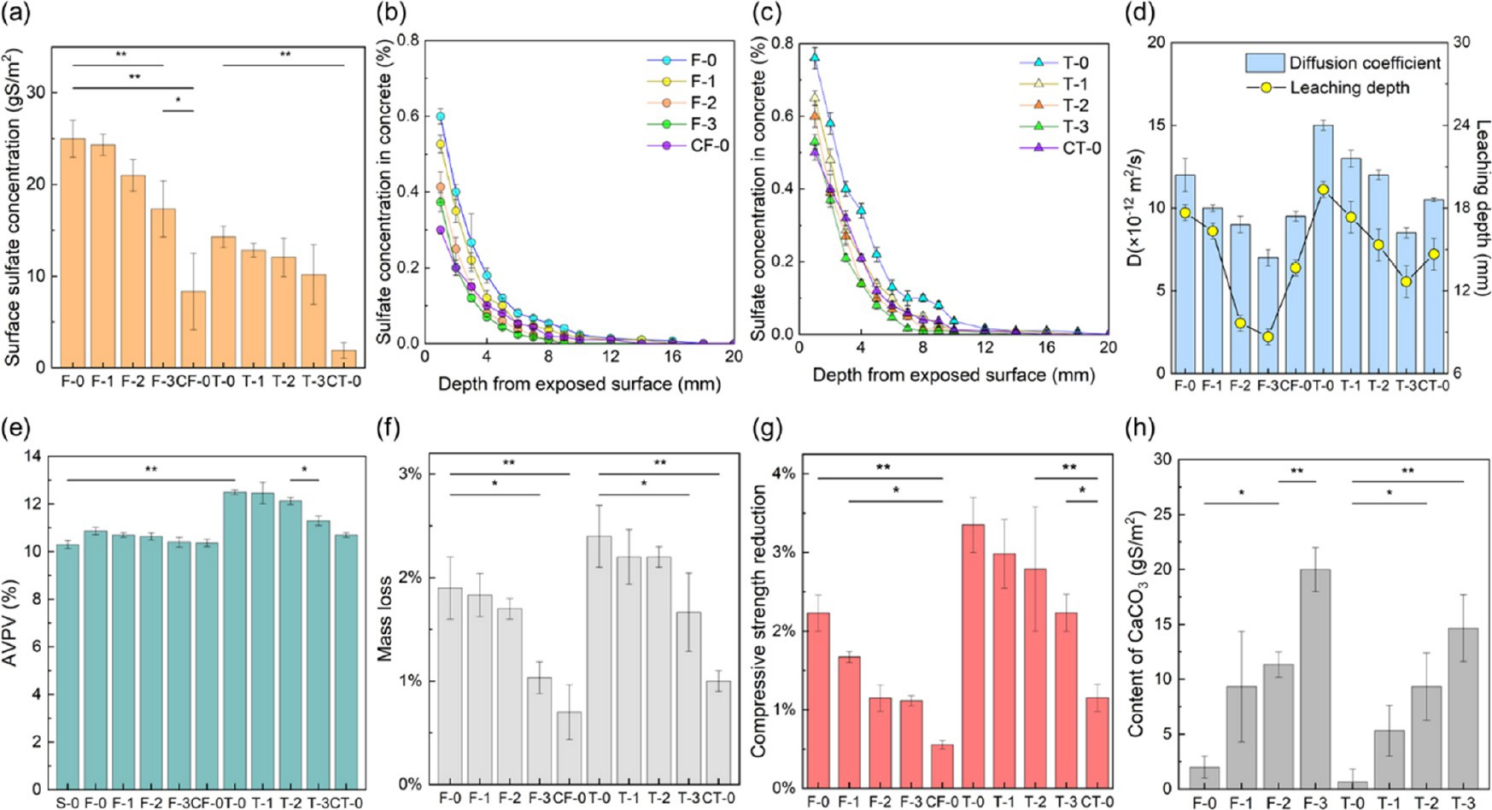
Comparison of sulfur levels after corrosion: (a) sulfate on the surface of the concrete; (b) sulfate concentrations in concrete for the submerged groups; and (c) sulfate concentrations in concrete for the tidal groups. Panel (d) is the diffusion coefficient and leaching depth obtained based on the sulfate concentrations at different depths in concrete. After corrosion, the change in the (e) apparent volumes of permeable voids (AVPV), (f) concrete mass, and (g) compressive strength, respectively. Panel (h) is the content of CaCO_3_ on the surface of the concrete for the specimens with MIC and BCCI. The error bar represents the standard deviations (** indicates a highly significant difference, *p* < 0.01 and * indicates a significant difference, *p* < 0.05).

The surface sulfate concentration of F-0 was the highest (Figure 2a). Comparably, the surface sulfate concentrations of F-3 and CF-0 were 43.3 and 56.6% less than that of F-0. The tidal groups had lower surface sulfate concentrations than the submerged groups due to a higher surface pH. A highly significant difference was detected between T-0 and CT-0 (*p* < 0.01). Biomineralization resulted in a decrease in the production of sulfuric acid. Surface sulfate concentrations decreased with increased UPB concentrations, further increasing the pH. The pH of the system plays an important role in biofilm adhesion; a higher pH cannot create a favorable environment for the growth of microorganisms and inhibits the adhesion and growth of corrosive microorganisms,^36^ leading to less sulfuric acid produced from them. Overall, the higher surface pH from biomineralization was beneficial for mitigating corrosion due to both a decrease in the production of sulfuric acid and an inhibition in the growth of microorganisms.

A previous study reported that microbial activity can spread across the deterioration zone rather than just at the layers of corrosion close to the surface.^58^ The microbes in the deterioration zone accelerated their penetration directly into the concrete further weakening the internal concrete structure.^59^ The sulfate concentrations in the MIC samples (F-0 and T-0) were indeed much higher than those in the CC samples for both groups (Figure 2b,c); therefore, the MIC samples had larger diffusion coefficients and leaching depths (Figure 2d). Biomineralization effectively decreased the sulfate concentrations in the concrete. This suggests that the biomineralized film acted as a protective layer controlling the diffusion of sulfate, leading to smaller diffusion coefficients and leaching depths. In addition to aggressive ion attack, the tidal groups also experienced alternating wetting-drying processes. High concentrations of sulfate can move from the surface to the inner layers of the concrete by diffusion and penetration due to the changes in the pressure gradient at the pores and cracks during the wetting-drying process.^60^ As a result, the internal sulfate concentrations in the tidal groups were higher than those in the submerged groups despite fewer surface corrosion products, contributing to larger diffusion coefficients and greater leaching depths.

In this study, the corrosion resulted in several small holes on the surface of the concrete (Figure S3), and the AVPV increased after corrosion (Figure 2e). Figure 2f, g shows that the mass loss and strength reduction decreased in the concrete, with MIC > BCCI > CC. The tidal inoculation resulted in a larger increase in AVPV than in the submerged inoculation because of the dual impact of microbes and the wetting-drying process. Compared to the submerged groups, there was also a larger reduction in the strength of the concrete in the tidal groups. Furthermore, the BCCI samples had relatively smaller AVPV than the MIC samples despite different inoculations. The more compact biomineralized film might have mitigated the generation of small holes or the UPB might have healed the surface cracks or pores,^26^ both of which could lead to a decrease in AVPV. The concrete with a high concentration of UPB was more corrosion-resistant, leading to a smaller mass loss or strength reduction. The changes in mass loss and strength reduction of a six-month corrosion experiment are shown in Figure S5, suggesting that the BCCI samples always had better resistance performance in the six-month experiment.

Some SRB communities can also induce the formation of CaCO_3_ precipitation;^61^ thus, we observed a small amount of CaCO_3_ in F-0 and T-0 (Figure 2h). The enriched UPB in biomineralization utilized CO_2_ to form CaCO_3_ precipitation on concrete surfaces; thus, about 10 times CaCO_3_ content was detected in F-3 and T-3 compared to F-0 and T-0, contributing to carbon neutrality (for the detailed results, please refer to Section S10).

### 3.3 Characterizations of the Morphology of Corrosion Products

**Figure 3:**
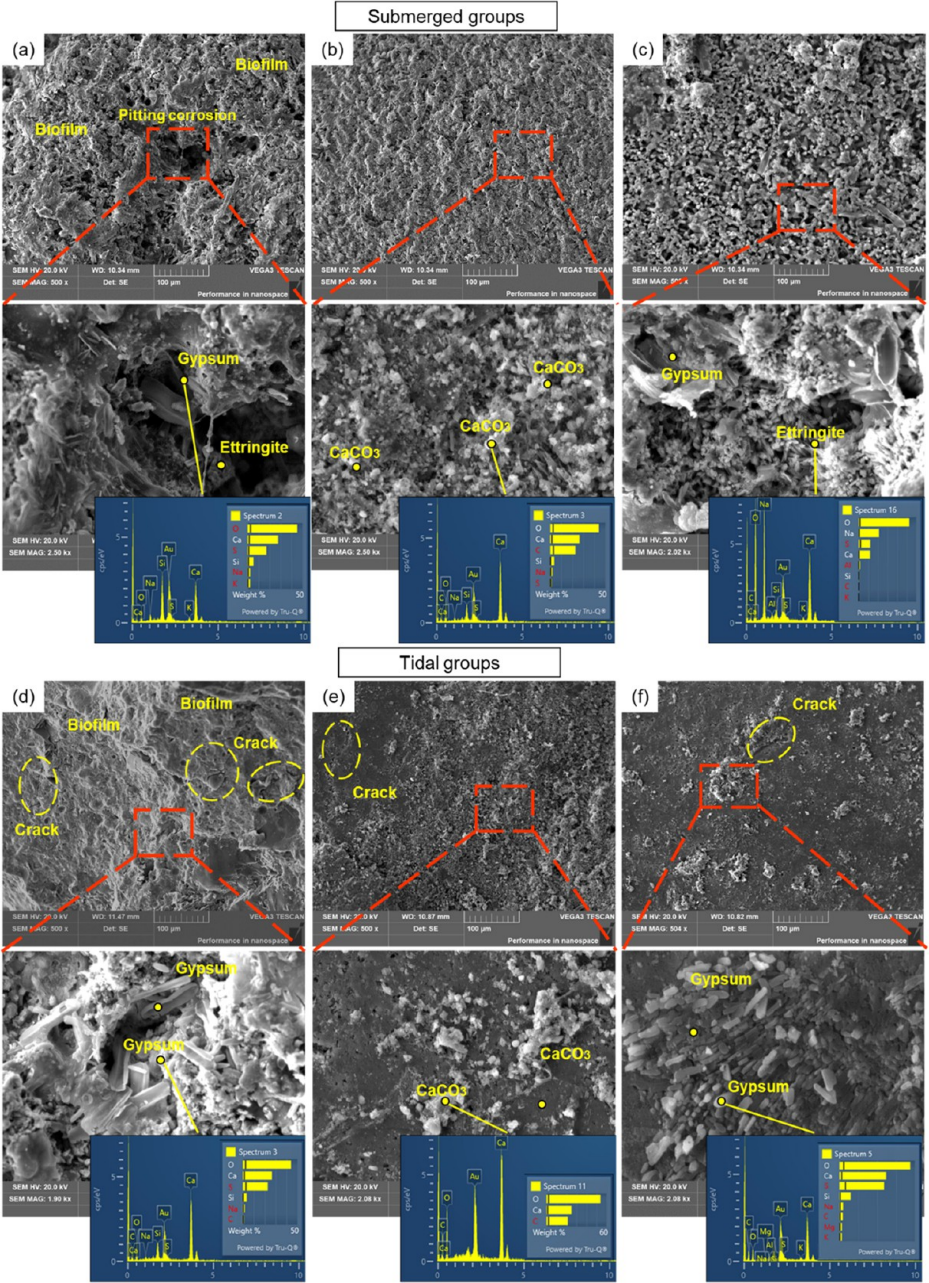
SEM and EDX images of the surface of the concrete with submerged inoculation: (a) MIC, (b) BCCI (F-3), and (c) CC; and with tidal inoculation: (d) MIC, (e) BCCI (T-3), and (f) CC.

For the submerged group, a dense and uneven three-dimensional structure of biofilm with microbes embedded on the MIC concrete surfaces was observed, as well as pitting corrosion (Figure 3a), which was quite different from that of the concrete surface before corrosion (Figure S6). In the areas of pitting corrosion, gypsum and ettringite had formed, which were confirmed by XRD analysis (Figure S7). With regard to the BCCI concrete, however, the biomineralized film on the concrete surface was uniform and compact, and a large number of precipitated CaCO_3_ crystals were produced (Figure 3b). The CaCO_3_ that precipitated via biomineralization was often identified as calcite.^26,62^ The CaCO_3_ crystals in the biomineralized film were rhombohedral and were also related to calcite. For the CC concrete, gypsum and ettringite were also produced on the concrete surface (Figure 3c). As for the tidal groups, biofilms with microbes and gypsum were observed on the MIC concrete surface (Figure 3d), and the biomineralized film with CaCO_3_ precipitation formed on the BCCI concrete surface (Figure 3e). The CC concrete in the tidal groups also experienced corrosion (Figure 3f), but the quantity of gypsum was much smaller than that on the MIC concrete surface. Several cracks were also noted on the concrete surfaces in the tidal groups, which were mainly attributed to the wetting-drying process.

### 3.4 Composition and Succession of Microbial Communities in Concrete Biofilms

**Figure 4:**
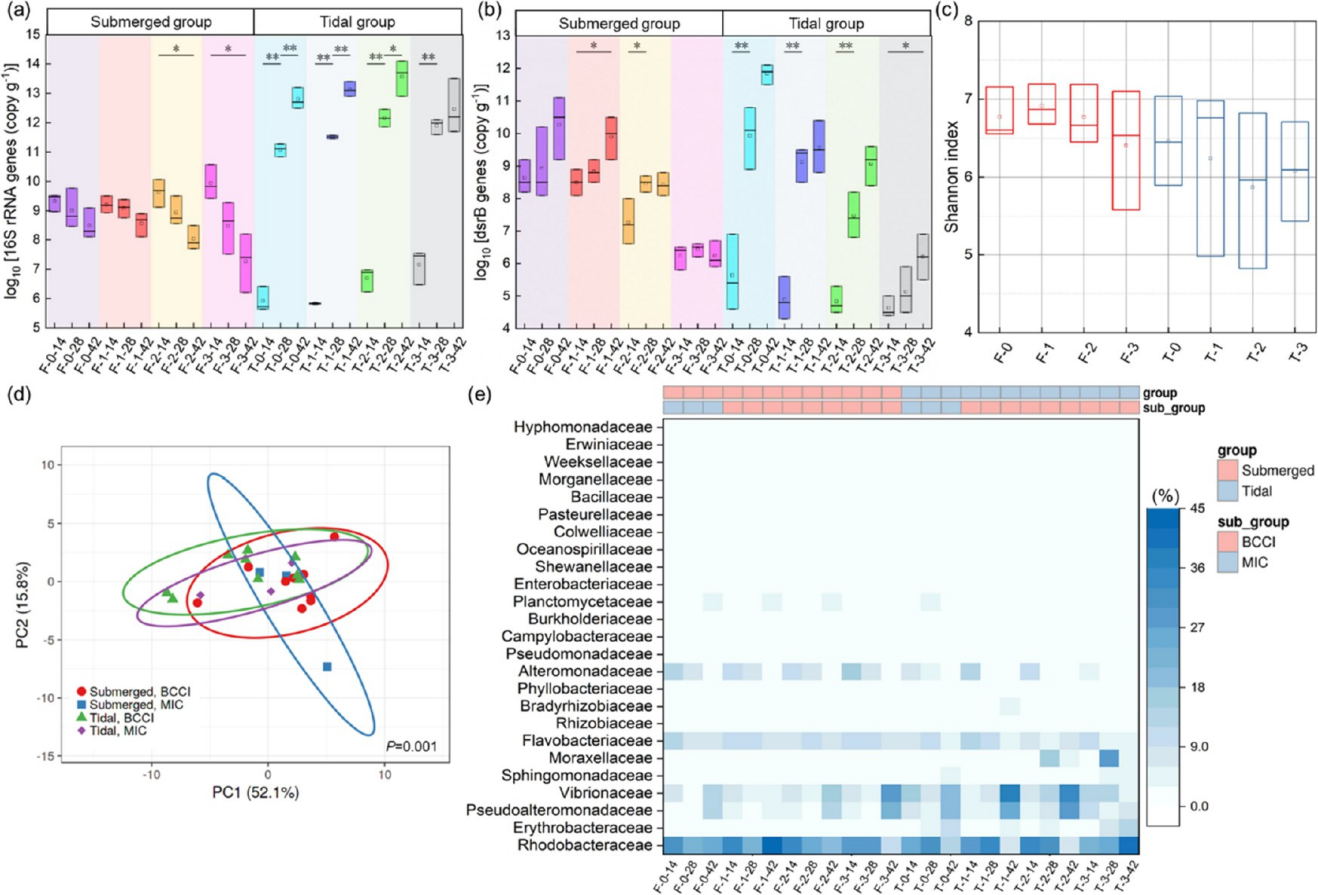
qPCR analysis of evolution for concentrations (copy g^–1^) of (a) 16S rRNA gene and (b) *dsrB* in concrete biofilms. Panel (c) is the α diversity of biofilm communities at the species level (Shannon index). Panel (d) shows differences in the microbial communities as visualized with PCoA. The permutational multivariate analysis of variance (PERMANOVA) was used to test differences in the microbial communities between the groups. *P* < 0.05 was regarded as the criterion for statistical significance in any differences. Ovals indicate the 95% confidence intervals for each sample type. Panel (e) shows the proportion of bacteria from concrete biofilms classified at the family level as being in the top 20.

On the 14th day, specimens with higher concentrations of UPB coating were not surprisingly observed with higher loads of 16S rRNA gene (an indicator of total bacteria) on the surface in both groups (F-3 and T-3) (Figure 4a) since UPB could be the dominant bacterial component on the concrete surface in the early stage of biofilm formation. For the submerged groups, the preadded UPB accelerated the succession of biofilms and the subsequent attachment of nonbacteria occurred earlier, which decreased the concentrations of bacteria. Therefore, the bacterial loads per gram gradually decreased over time. Such a phenomenon was more remarkable for samples with higher concentrations of UPB coating. Nevertheless, the concentrations of the 16S rRNA gene significantly increased from the 14th day to the 42nd day for all tidal groups. The tidal groups experienced a small effect of nonbacteria (Figure S12a), suggesting that the succession of biofilms was slower than the one in the submerged groups.

On the 14th day, the submerged groups had much higher concentrations of *dsrB* than the tidal groups (Figure 4b), which indicated that the submerged inoculation caused more severe corrosion from the SRB communities. The covering of the surface by corrosion products can limit SRB from accepting the electrons needed for their metabolic processes.^63^ The covering of the biomineralized film also limited SRB; thus, the BCCI samples had a lower load of *dsrB* than the MIC samples. For F-2 and F-3, *dsrB* concentrations increased from the 14th day to the 28th day and then slightly decreased after 28 days because the mature biomineralized film effectively limited the growth of SRB. For the tidal groups, the concentrations of *dsrB* steadily increased from the 14th day to the 42nd day. However, compared with that in MIC samples, the rate of increase was much smaller in the BCCI samples, especially in T-3. This suggested that the biomineralization still had larger inhibiting effects, especially even after 28 days. The results demonstrated that biomineralization enabled a significant decrease in the total abundance of SRB (details of qPCR results are presented in Figures S8 and S9 in Section S13).

The microbial communities in the submerged groups were more diverse than those in the tidal groups (Figure 4c). Different from the submerged samples, the biofilm in the tidal groups was in an initial development stage, and the increase in UPB concentration made UPB more dominant in the biofilms, resulting in a slightly less diverse microbial community in the tidal groups. Figure 4d shows a smaller change in the community structure of the BCCI samples in the submerged groups than in the MIC samples. The difference in the bacterial communities between the MIC and BCCI samples gradually decreased as well as the difference between the submerged and tidal groups (Section S14 and Figure S10).

The bacterial communities in concrete biofilms were numerically dominated by *Proteobacteria* and *Bacteroidetes* (Figure S11a). The dominant bacterial phyla were different from the concrete biofilms in river environments (*Proteobacteria* and *Actinobacteria*) reported in the literature.^44^ Previous studies on the bacterial communities of biofilms also reported that *Proteobacteria* dominated in many marine environments.^64−67^ It was apparent that the dominant bacterial communities gradually changed as corrosion progressed due to the changed environmental conditions (e.g., surface pH and oxygen) (Figure 4e). The family *Rhodobacteraceae* was highly abundant in all of the concrete biofilms. There was a larger proportion of the genus *Sulfitobacter*, which was related to sulfur^68^ from the family *Rhodobacteraceae*, and it was identified as one of the top 20 genera (Figure S11b). The members of this family, such as the chemoorganotrophic bacterium *Sulfitobacter* sp.THAF37, *Sulfitobacter* sp.D7, and *Sulfitobacter pseudonitzschiae*, were identified as being among the top 20 species (Figure S11c).

### 3.5 Profiles of Corrosive Bacterial Communities in Concrete Biofilms

**Figure 5:**
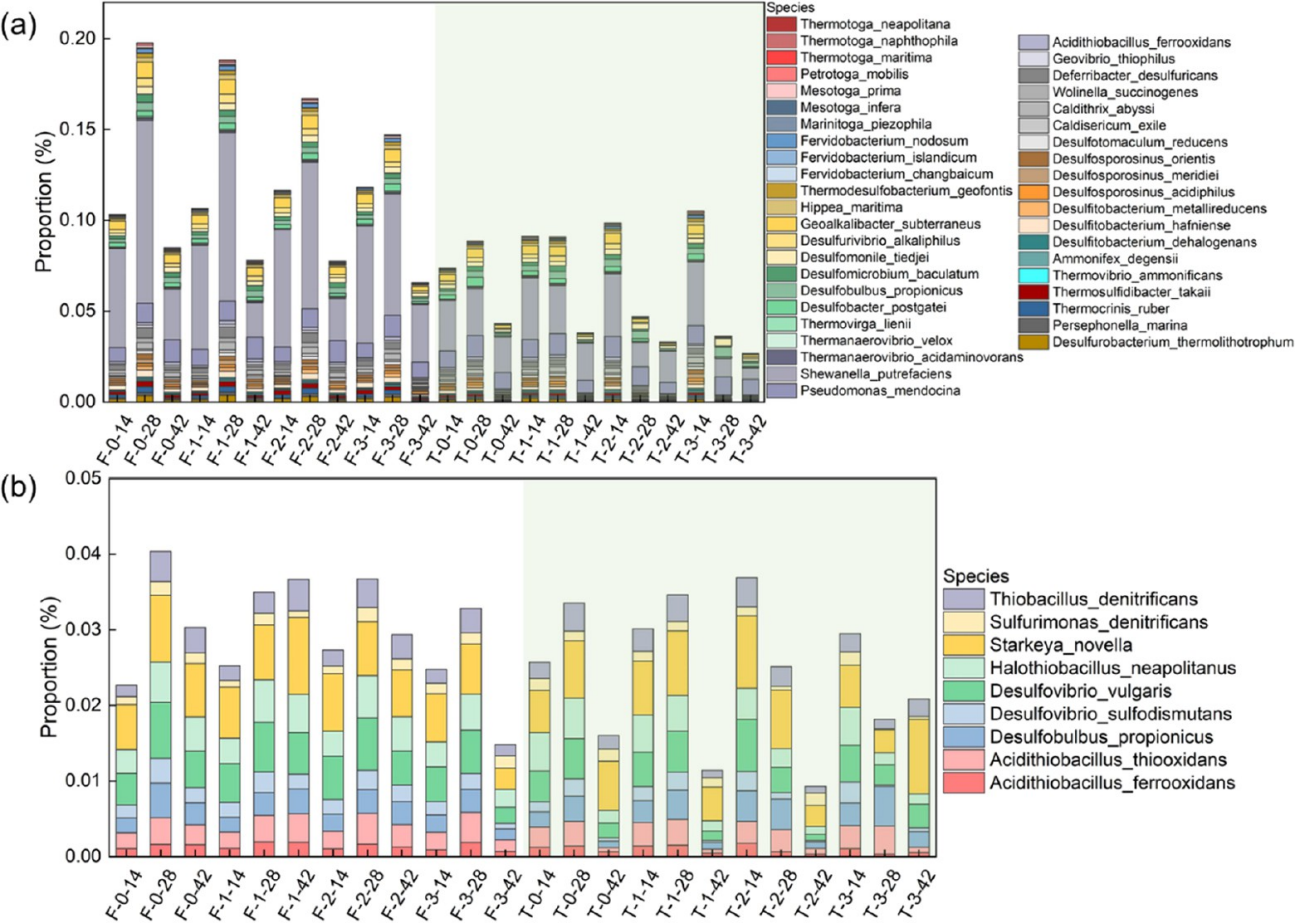
Changes in (a) SRB profiles and (b) SOB profiles in different concrete biofilms, respectively.

SRB communities have been confirmed to suffer under highly alkaline conditions.^69^ In this study, however, the alkalinity of seawater might favor the attachment and growth of these strains on concrete surfaces due to their better alkalinity resistance. The proportions in the submerged groups were much higher than those in the tidal groups (Figure 5a). The rapid growth of UPB in the biomineralized film competed with SRB for nutrients and colonized sites;^22^ therefore, the total abundances of SRB in the BCCI samples were always lower than that in the MIC samples (Figure 4b). Furthermore, the formation of the biomineralized film removed oxygen, greatly inhibiting the growth of aerobic bacteria; however, most SRB can survive in anaerobic environments;^70^ thus, a higher relative abundance of SRB for the BCCI samples than for the MIC samples was detected on the 14th day, regardless of the group (Figure 5a). From the 14th day to the 28th day, the proportion of SRB in the submerged groups increased and the data for the MIC samples exceeded the BCCI samples, suggesting that the formation of the biomineralized film effectively inhibited the colonization and growth of SRB communities. Due to the short incubation period in our present investigation, bacterial taxa were more heavily represented in the concrete biofilms than eukaryotic taxa (Figure S12a). More eukaryotic taxa subsequently colonized the concrete surface in the submerged groups (Figure S12b). The increases in the proportions of several eukaryotic phyla (e.g., Basidiomycota, Ascomycota, and Diatomea) might account for increased DO concentrations.^71−73^ This might have accelerated the growth of competitive aerobic bacteria and decreased the proportion of SRB on the 42nd day. For the tidal group, the tidal condition provided sufficient oxygen for the growth of aerobic bacteria. T-0 also followed this trend because the aerobic bacteria competed with SRB, decreasing its proportion after 28 days. However, the proportion of SRB in the BCCI samples invariably decreased, especially T-3, because the initial SRB communities were inhibited by the formation of the biomineralization film.

In addition to SRB, SOB also play an important role in sulfur cycles, which can be categorized into neutrophilic SOB for an initial sulfuric acid attack, and acidophilic SOB for a further severe acid attack.^74^ In this study, neutrophilic SOB (e.g., *Starkeya novella* and *Halothiobacillus neapolitanus*) were always much more abundant than acidophilic SOB (e.g., *Acidithiobacillus ferrooxidans* and *Acidithiobacillus thiooxidans*), indicating that the concrete samples had experienced an initial attack of sulfuric acid and were under severe acid attacks within the first 2 weeks of the experiment (Figure 5b). However, the second stage appeared to develop slowly since there was little fluctuation in the proportion of acidophilic SOB throughout the experiment. Two major different pathways for thiosulfate oxidation were confirmed between acidophilic SOB and neutrophilic SOB.^4,75^ The difference in the diversity in function between the two types of SOB in concrete biofilms may be the main reason for the different corrosion rates in different MIC environments (e.g., seawater, sewage structures, and wastewater treatment plants).^8,76^ Different from SRB, there was no clear difference in SOB abundance in concrete biofilms between the submerged and tidal groups nor a difference between the MIC and BCCI samples due to the low SOB abundance in a short incubation period. In the initial succession period of biofilms, SRB communities were more responsive than SOB communities to concrete corrosion (for details of corrosive bacterial communities, please refer to Section S14). For the tidal experiment groups, less biofilms attached to the concrete surface and the low relative abundance of SRB eventually contributed to fewer surface corrosion products, higher pH values, and lower concentrations of surface sulfate.

### 3.6 Influence of MIC on Marine Concrete Structures

**Figure 6:**
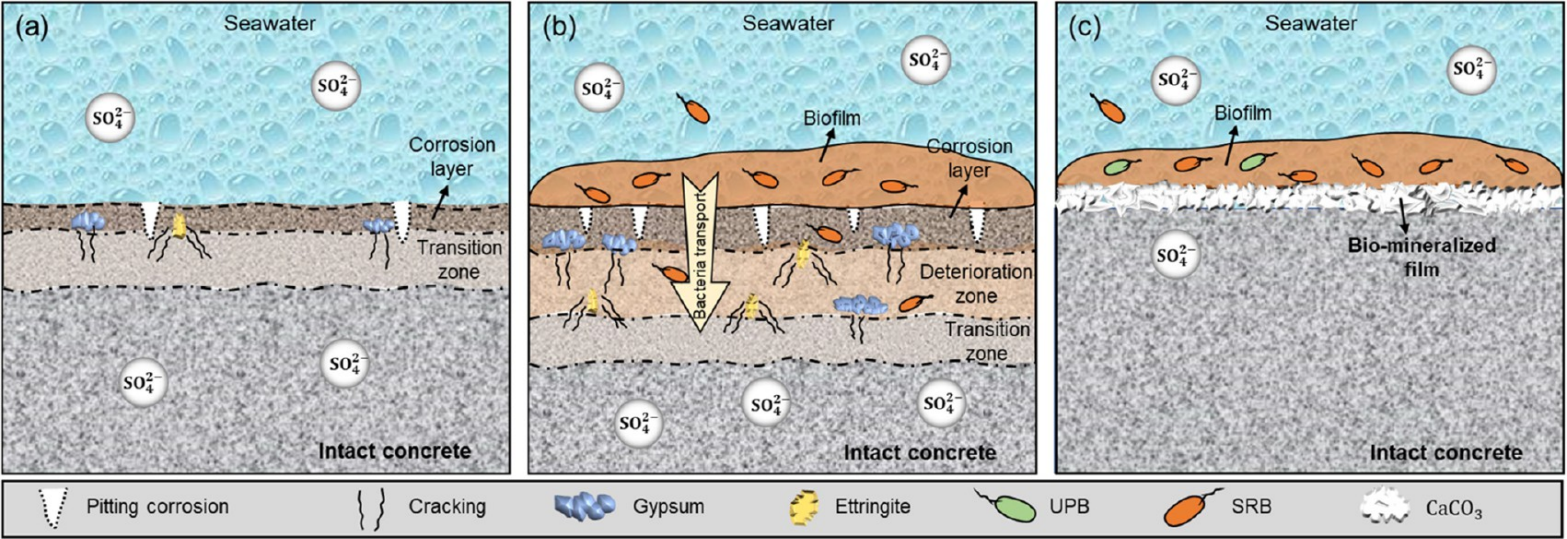
Corrosion mechanism for (a) chemical corrosion; (b) biofilm corrosion; and (c) biomineralization for corrosion inhibition.

When concrete comes into contact with SO_4_^2–^ in Na_2_SO_4_ in seawater, calcium hydroxide and calcium aluminate hydrate will be consumed to form gypsum and ettringite, resulting in expansion stress and matrix fracture^17,77,78^ (Figure 6a). In a pure sulfuric acid attack, the corrosion layer (mainly consisting of gypsum) can act as an extra barrier that inhibits acid penetration.^79,80^ However, the role of the corrosion layer can be very different in an MIC attack. In an MIC attack, bacteria can colonize the corroded layer, which provides an excellent medium for microorganisms to grow.^57^ Microbial activity can spread across the deterioration zone rather than just limit in the corrosion layer close to the surface.^58^ As the corrosion progresses, corrosive bacteria would proceed further along the resulting cracks and colonize into the deeper layers.^26^ Therefore, there was a stratification phenomenon, causing different conditions at different depths. For MIC, the surface pH quickly decreased due to the production of sulfuric acid, accelerating the corrosion and diffusion of sulfate (Figure 2c, b). Figure 6b shows more gypsum and ettringite formation in the deteriorated zone. Aerobic and facultative microbes in the top layer of the biofilms can provide a locally oxygen-free environment for SRB communities to grow;^63,81^ therefore, the corrosion would become more severe over time. The functional prediction can be used in future studies to obtain a mechanistic understanding of the potential metabolic capability of microbial action on concrete corrosion, which is beneficial for unraveling the black box between SRB and the lifespan of marine concrete structures.

Moreover, the corrosion rates (mass loss and strength reduction) collected from previous studies were compared with the corrosion rates in this study (Section S15 and Table S9). A comparison of the results suggests that submerging concrete in seawater would also result in severe corrosion (Figure S13). Many researchers have suggested that the highly alkaline surface of concrete is the fundamental reason why microbes find it difficult to colonize concrete surfaces.^82^ However, the alkalinity of seawater makes the colonization of microbes easier and causes serious MIC issues for marine concrete structures. Therefore, the ratios of mass loss rates between MIC and CC in this study reached 2.71 or 2.40 (the submerged groups and the tidal groups) and 3.99 or 3.00 for the ratios of strength reduction rates (Section S16 and Table S10). As with exposure to the sewer environment,^11,57,83^ corrosive microorganisms significantly accelerate corrosion and cut the lifespan of marine concrete structures from an expected service life of 100 years down to 40–50 years and even to 30 years or fewer. We should pay more attention to the MIC on marine concrete and to the overestimations of the lifespans of marine concrete structures.

### 3.7 Environmental Implications

Protective biofilms can provide a barrier to inhibit corrosion.^19,84^ In relation to this, a promising research direction is to improve the compactness of the biofilm to completely isolate the surface from the corrosive medium. In this study, the biomineralization approach was proposed for inhibiting concrete corrosion. Compared to CC, MIC caused much higher corrosion rates and would significantly reduce the lifespan of marine concrete structures. However, the formation of the biomineralized film on the concrete surfaces effectively decreased the abundance of SRB in overall biofilms, leading to higher surface pH and lower surface sulfate concentrations. The biomineralized film also acted as a protective layer to control the diffusion of sulfate and isolate the concrete from SRB communities, decreasing internal sulfate levels (Figure 6c). A higher concentration of UPB had a better inhibition performance (lower mass loss and strength reduction) than most previously reported methods for inhibiting concrete corrosion (using AAC, CAC, or adding SCMs) (Section S17 and Table S11). Moreover, the type of colonized surface also affects the inhibition performance of biomineralization because dominant taxa may differ with the type of colonized surface,^85^ leading to different rates of corrosion for different types of concrete. The proposed method is promising and deserves further study.

The biofilm structure at the species levels changed with the UPB concentration in the tidal groups, while there was no significant difference between the MIC and BCCI samples in the submerged groups (Figure S10). This was because biofilms formed in layers and matured over time; the structure of the biofilm community tended to be similar at the surface layer, despite the presence of different microbial communities at the bottom layer. For the submerged group, more mature and similar community structures were observed in the MIC and BCCI samples. The tidal groups also had increasingly similar biofilm communities from the 14th day to the 42nd day. Although the initial formation of the biomineralized films resulted in different initial bacterial communities, the subsequent similar communities colonized on top of the existing biomineralized film eventually led to the bacterial community structures that were increasingly similar to the naturally formed biofilm on the MIC samples. The results indicate that the biomineralization technique is environmentally friendly and has a low impact on the overall biofilm communities as a coating method for controlling concrete corrosion. In addition, as biomineralization for corrosion inhibition utilizes CO_2_ to produce precipitates to improve the durability of concrete structures, this process will reduce the carbon footprint and energy consumption of marine infrastructure during the stage of its use and contribute to carbon neutrality and sustainability.

This biomineralization strategy has strong potential to be applied to corrosive environments, such as marine environments, sewage environments,^14^ and water cooling utilities,^81^ where concrete corrosion is induced by corrosive microorganisms.

## Supporting Information Available

Formula for making SSC, the enrichment of UPB in seawater, arrangements to conduct the laboratory concrete corrosion experiments, the qPCR procedure, measurements of mechanical property of concrete, SRB and SOB communities in previous studies, summary of results, the analysis of the physicochemical parameters of seawater, the analysis of the genotypic data of biofilms, the detailed profiles of corrosive bacterial communities, corrosion rates in previous studies, and inhibition effects of different inhibition strategies on concrete corrosion (PDF)
