# Supplementary material for: Biomineralization To Prevent Microbially Induced Corrosion on Concrete for Sustainable Marine Infrastructure
Source: Environ Sci Technol. 2023 Dec 5;58(1):522–33. doi: 10.1021/acs.est.3c04680 (PMC10785763; doi:10.1021/acs.est.3c04680)
Supplement: Supplementary file 1 — es3c04680_si_001.pdf [file es3c04680_si_001.pdf]

Supporting Information

Biomineralization to prevent microbially induced corrosion on concrete for sustainable marine infrastructure

Xiaohao Sun[a], Onyx W.H. Wai[a,b], Jiawen Xie[a], Xiangdong Li[a,b*]

[a] *Department of Civil and Environmental Engineering, The Hong Kong Polytechnic University, Hung Hom, Kowloon, Hong Kong SAR, China*

[b] *Research Institute for Sustainable Urban Development, The Hong Kong Polytechnic University, Hung Hom, Kowloon, Hong Kong SAR, China*

Xiaohao Sun (xiaohsun@polyu.edu.hk)

Onyx W.H. Wai (ceonyx@polyu.edu.hk)

Jiawen Xie (jiawen.xie@connect.polyu.hk)

*Corresponding author

Email: cexdli@polyu.edu.hk

Tel: (852) 2766-6041

Fax: (852) 2334-6389

This supporting information contains 17 sections, 13 figures, and 11 tables (62 pages in total)

Contents

Supporting Information....................S1
Biomineralization to prevent microbially induced corrosion on concrete for sustainable marine infrastructure....................S1
Section S1. SSC making formulation....................S5
Table S1. Mix proportions of SSC (kg/m^3)....................S5
Section S2. Enrichment of urease-producing bacteria (UPB) in seawater....................S6
Table S2. Experiment arrangement for the enrichment of UPB in seawater....................S6
Figure S1. (a) Evolution of urea concentration and (b) the variation of viable cell concentration during enrichment; (c) the change of urease activity with cultivation time; and (d) solution urea concentration measured in the biomineralization process. S7
Section S3. Laboratory concrete corrosion experiment....................S10
Table S3. Concrete specimen arrangement for corrosion experiment....................S10
Section S4. Physicochemical parameters analysis of seawater....................S12
Table S4. Physicochemical parameters of seawater sampled from the Tsim Sha Tsui Pier every week....................S12
Table S5. Physicochemical parameters of the seawater in the water tank every week....................S14
Section S5. Measurements of surface sulfate concentrations and $CaCO_3$ contents....................S16
Section S6. Measurements of mass loss, AVPV, strength reduction, and sulfate concentrations in concrete....................S17
Section S7. qPCR procedure....................S19
Section S8. SRB communities....................S21
Table S6. Commonly studied SRB in marine corrosion....................S21
Section S9. SOB communities....................S24
Table S7. Commonly studied SOB in concrete corrosion....................S24
Section S10. Summary of the results....................S25
Table S8. Summary of the results of the laboratory concrete corrosion experiment....................S25
Section S11. Physicochemical parameters and genotypic data analysis of seawater during corrosion....................S30

Figure S2. The evolution during the experiment for (a) the temperature of seawater in the water tank and atmospheric temperature; (b) the pH and (c) the DO of the seawater in the water tank. S30

Figure S3. The concrete surface of F-0 after 14-day corrosion........ S31

Figure S4. qPCR analysis for concentrations of (a) the 16S rRNA gene and (c) dsrB in the seawater sampled from the Tsim Sha Tsui Pier, and concentrations of (b) the 16S rRNA gene and (d) dsrB in the seawater in the water tank. S32

Section S12. Mechanical properties, morphology characterizations, and corrosion products S34

Figure S5. After the six-month corrosion, the change in (a) concrete mass and (b) compressive strength, respectively. The BCCI samples had the same biomineralization treatment condition as the F-3 and T-3 in Figure 2. .... S34

Figure S6. The SEM image of concrete without corrosion S35

Figure S7. The XRD results of corrosion products: (a) the submerged groups; (b) the tidal groups. S35

Section S13. qPCR results of biofilm on concrete surface S37

Figure S8. Evolution of concentrations of specific gene contents in concrete biofilms quantified by qPCR: (a) 16S rRNA gene after 14 days; (b) dsrB after 14 days; (c) 16S rRNA gene after 28 days; (d) dsrB after 28 days; (e) 16S rRNA gene after 42 days; (f) dsrB after 42 days. S37

Figure S9. Comparison of Chl-a concentration for different concrete specimens after corrosion. S40

Section S14. Genotypic data analysis of biofilms S41

Figure S10. Microbial community differences as visualized with PCoA on (a) the 14th day, (b) the 28th day, and (c) the 42nd day. The permutational multivariate analysis of variance (PERMANOVA) was used for tests of inter-group differences. P < 0.05 was regarded as the criterion for statistical significance of the difference. Ovals indicate the 95% confidence intervals for each sample type. S41

Figure S11. Dominant bacterial composition in concrete biofilms: (a) top 10 at the phylum

level, (b) top 20 at the genus level, and (c) top 20 at the species level. S42
Figure S12. Comparison of biofilm profile during the corrosion experiment at the (a) kingdom and (b) phylum levels. S44
Section S15. Different corrosion rates in different corrosion experiments....................S46
Table S9. Comparison of corrosion rates in different experiments S46
Figure S13. Comparison of corrosion rate between the current study and previous studies: (a) based on both the mass loss and strength reduction; (b) only based on mass loss; and (c) only based on strength reduction. S50
Section S16. The influence of bacteria on concrete corrosion...S53
Table S10. Comparison of microbially induced corrosion and chemical corrosion S53
Section S17. Inhibition effects of different inhibition strategies on concrete corrosion S54
Table S11. The corrosion inhibition effects of various methods S54
References...S56

Section S1. SSC making formulation

Table S1. Mix proportions of SSC (kg/m^3)

Materials	Material Type and Source	Quantity per m^3 Concrete (kg)	Specific Gravity (g/cm^3)	Volume (m^3)
Cement	Ordinary Portland Cement, Japan	330	3.15	0.105
PFA	Pulverized Fuel Ash, Hong Kong	110	2.20	0.050
20 mm Agg.	Crushed Rock, Guangdong, China	565	2.62	0.216
10 mm Agg.	Crushed Rock, Guangdong, China	465	2.62	0.177
Sea sand	Sea sand, Guangdong, China	730	2.62	0.279
Sea Water	Sea Water, near Tsing Yi Island	165	1.02	0.162
Admixture	Rheomac 1002, Superplasticizer ex. BASF Hong Kong Limited	2.65±2.12	1.06	0.003
	Total	2370	-	0.992

The sea sand had a particle size not exceeding 1.18 mm. The concrete specimens had a slump of 125 mm and a water/cementitious ratio by weight of 0.38.

Section S2. Enrichment of urease-producing bacteria (UPB) in seawater

Table S2. Experiment arrangement for the enrichment of UPB in seawater

Sample NO.	Yeast extract concentration (g/L)	Urea concentration (mM)	Ammonia chloride (mM)
Y10U5	10	50	50
Y20U5	20		
Y10U10	10	100	
Y20U10	20		

Several enrichment media were prepared for the enrichment of UPB in the seawater sampled near the Tsim Sha Tsui Pier in Victoria Harbour. Yeast extract, a common nutrient for the growth and reproduction of UPB, was added to 100 mL seawater at two concentrations to study the influence of nutrients on the enrichment effect (Table S2). Moreover, the urea added to the enrichment media also varied (50 mM and 100 mM). These media with an adjusted pH of 8.0 were then put in a shaker at 200 rpm and 25 °C for 48 h. The urea concentration was determined spectrophotometrically during the enrichment by measuring the absorbance at 425 nm wavelength at 0, 8, 12, 24, and 48 h [1] (Figure S1a). Meanwhile, isolates were collected; the plate counting method was used to obtain the cell densities [2] (Figure S1b) and the urease activity was monitored at 0, 8, 12, 24, and 48 h [3] (Figure S1c). To comparatively study ureolytic capacity related to biomineralization of these media after enrichment, 20 mL bacterial suspension cultured for 36 h was mixed with 20 mL urea solution (500 mM) at 2 5 °C. The urea concentration was measured every day to comparatively study the ureolytic capacity of enriched UPB in different media (Figure S1d), to obtain the optimum growth medium for subsequent studies.

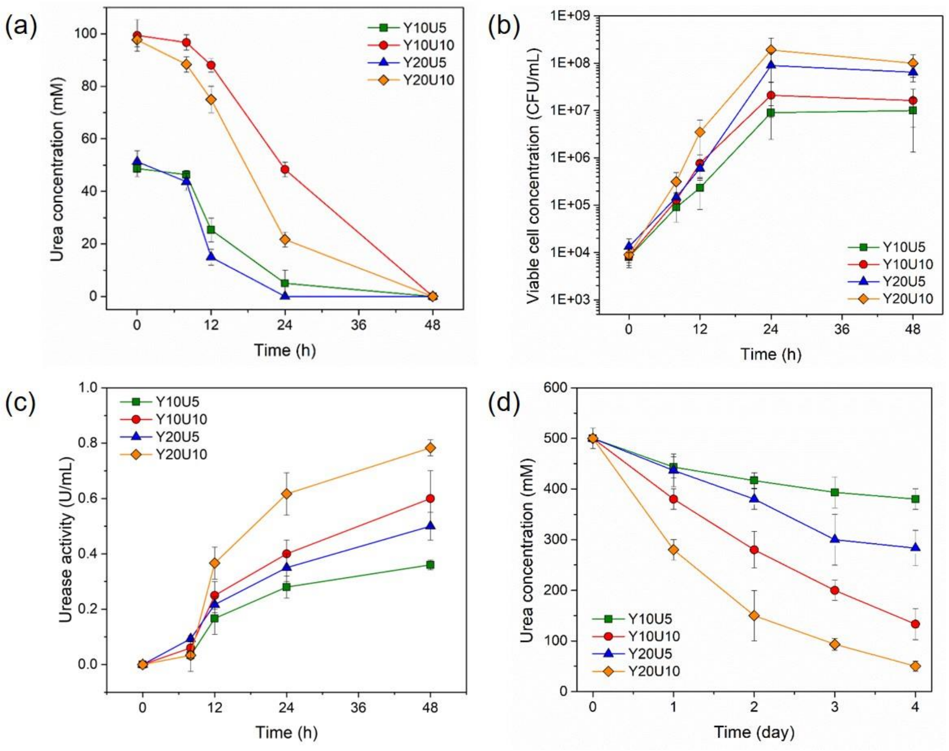

Figure S1. (a) Evolution of urea concentration and (b) the variation of viable cell concentration during enrichment; (c) the change of urease activity with cultivation time; and (d) solution urea concentration measured in the biomineralization process.

The media Y20U10 and Y20U5 always had larger viable cell concentrations due to more nutrients in the media. During enrichment, the UPB can decompose urea in media into carbonate ions and ammonium ions. Therefore, urea in media is a very important component for the enrichment of UPB [2, 4]. The urea concentration gradually decreased with the growth and reproduction of UPB. The initial ureolytic capacity was minimal with scarcely detectable urea degradation over the 8 h, regardless of media. However, at 24 h, the remaining urea concentration decreased to below 60 mM, especially the Y20U10, strongly suggesting that significant enrichment of UPB took place from 8 h to 24 h. The decrease in urea concentration can result in a decreased urea hydrolysis rate [5]. Therefore, there was a smaller decreasing rate

of urea concentration after 24 h, despite a higher cell density. The urea concentrations in the four yeast extract media all decreased to zero after 48 h. During the enrichment, the media Y20U10 always showed the larger urease activity. A lower urea concentration contributed to the lower urease activity. Moreover, the urea concentration might have a larger influence on the enrichment of UPB than the nutrient concentration, which can be concluded by comparing the urease activities between Y10U10 and Y20U5. It was because urea directly stimulated the growth of UPB, and the growth rate of UPB increased with an increase in urea concentration. The increase in nutrient concentration activated the growth of all kinds of bacterial strains, which lacked the targeting of UPB. The media Y10U5 had the smallest urease activities due to both the lower urea concentration and lower nutrient concentration.

Eventually, the media Y20U10 had the largest urease activity after enrichment, which resulted in the largest initial urea hydrolysis rates compared to the media with the identical nutrient used for bio-simulation, and the urea concentrations were always the lowest (Figure S1d). After only 1 day, the urea concentration decreased to about 280 mM in the media Y20U10. Subsequently, the urea concentration decreased, and the decreasing rate became lower and lower. The decreasing range of urea concentration from the 3rd day to the 4th day was quite small because the lack of nutrients resulted in the decay and death of UPB. The urea hydrolysis rate in this study was a little smaller than that of bacterial strains bio-simulated from the soil in most previously reported studies [6-8]. However, the clear decrease in urea concentration still indicated that the method employed here to enrich UPB in seawater was successful. Moreover, the media Y10U10 had larger urea hydrolysis rates than the media Y20U5 despite lower viable cell

concentrations. It indicated that a high urea concentration easily made UPB predominant with a strong ureolytic capacity. The UPB in Y10U10 accounts for a larger percentage of total bacterial strains than in Y20U5.

Due to sufficient ureolytic capacity, the UPB enriched under the growth medium Y20U10 (20 g/L yeast extract, 100 mM urea, and 50 mM ammonium chloride) was used for subsequent experiments by transferring 10% of the enriched culture into the optimized fresh growth medium to produce sub-culture, where agar was added to improve the coating adhesion of bacterial suspension on concrete surfaces. This sub-culturing was repeated multiple times to ensure the stability of urease activity [3].

The enriched UPB can break down urea to carbonate (CO_3^{2-}), which can bind with calcium ions in seawater to produce $CaCO_3$ crystals and form the biomineralization film. Some previous studies directly used the urea in seawater and successfully obtained the biomineralization film on the surface of materials without adding additional urea or calcium. It was because different from repairing concrete cracks, forming a thin biomineralization film does not need a high level of urea and calcium. These studies suggested that seawater has urea at a level supporting this process [9-11]. In our study, the cementation solution (1.0 M urea-calcium chloride solution) was added to quickly form a biomineralization film on concrete surfaces. Therefore, concrete surfaces were coated with a mixture of 12 mL bacterial suspension and cementation solution of 12 mL. These concrete specimens were kept at room temperature (20-25 °C) for 48 h to allow a complete reaction.

Section S3. Laboratory concrete corrosion experiment

Table S3. Concrete specimen arrangement for corrosion experiment

Sample No.	Inoculation	Treatment	The concentration of enriched bacterial suspension (CFU/mL)	Note
F-0	Submerged	Microbially induced corrosion (MIC)	/	Corrosion effect with naturally formed biofilm
T-0	Tidal		/	
F-1	Submerged	Biomineralization for concrete corrosion inhibition (BCCI)	~1×10^6	Effect of concentrations of bacterial suspension on corrosion inhibition
T-1	Tidal		~1×10^6	
F-2	Submerged		~5×10^6	
T-2	Tidal		~5×10^6	
F-3	Submerged		~1×10^7	
T-3	Tidal		~1×10^7	
CF-0	Submerged	Chemical corrosion (CC)	/	Removing microorganisms
CT-0	Tidal		/	
S-0	/	No corrosion	/	Original strength

An LED lamp was used daily from 9 am to 5 pm to simulate sunlight [12]. Real seawater used for the experiment was sampled from the Tsim Sha Tsui Pier in Hong Kong's Victoria Harbour. During the experiment half of the seawater in the water tank was changed every 7 days [10].

The flow velocity of the seawater in the water tank was set at about 20-35 cm/s based on marine data in the dry season, during which the overland runoff discharging into the coastal area is at a minimum [13-15]. The flow velocity affects the biofilm mainly due to a change in the dissolved oxygen (DO) concentration or shear stress [16-18]. The flow velocity might not be identical at different locations due to the non-uniformity of the flows, but DO concentrations were similar at different locations. In addition, with flow velocity increasing from 20 to 40 cm/s, the shear

stress caused a slight change to the biofilms [18]. As a result, the flow velocity may have a small impact on concrete corrosion at different locations.

Section S4. Physicochemical parameters analysis of seawater

Table S4. Physicochemical parameters of seawater sampled from the Tsim Sha Tsui Pier every week

Time	pH	Temperature (°C)	DO (mg/L)	Cond (ms/cm)	TOC (mg/L)	TN (mg/L)	NH3-N (mg/L)	BOD (mg/L)	Salinity (‰)	SO_4^{2-} (g/L)	Cl^- (g/L)	Chl-a (mg/m^3)
2022/1/12-0 week	7.89	18.2	6.03	37.2	6.89	0.3538	2.2	0.8	33	2.76	28.2	3.0
2022/1/19-1 week	7.58	17.8	5.86	39.4	6.17	0.2697	2	0.9	34	2.62	27.4	2.8
2022/1/26-2 week	7.85	18.6	5.03	42.1	6.32	0.4236	2	1.2	32	2.77	28.1	2.6
2022/2/2-3 week	7.55	17.2	6.31	35.6	7.04	0.4814	2.3	0.6	35	2.57	26.3	4.5
2022/2/9-4 week	7.58	18.4	5.05	42.4	6.65	0.2988	2	1.3	33	2.74	25.8	5.2
2022/2/16-5 week	8.01	18.7	5.18	38.7	6.72	0.6167	2.2	1.2	35	2.81	27.5	4.1

The physicochemical parameters included pH, temperature, dissolved oxygen concentration (DO), conductivity (Cond), total organic carbon (TOC), total nitrogen (TN), ammonium nitrogen (NH_3-N), biochemical oxygen demand (BOD), salinity, sulfate concentration (SO_4^{2-}), chloride concentration (Cl^-), and Chl-a (mg/m^3). The four exposure classes are (i) harmless (S0 for $SO_4^{2-} < 0.10$), (ii) moderate (S1 for $0.10 < SO_4^{2-} < 0.20$),

(iii) severe (S2 for 0.20 < SO_4^{2-} < 2.00), and (iv) very severe (S3 for SO_4^{2-} > 2.00) [19, 20]. In this study, exposure to seawater can be classified as very severe (S3).

The pH and temperature were measured using the pH meter with a Pt100 temperature sensor (pH-meter 605, Metrohm, Herisau, Switzerland). A mobile dissolved oxygen pocket meter was utilized to obtain the DO of seawater (Oxi340, WTW, Weilheim, Germany). The conductivity and salinity were measured with the conductivity meter (DDSJ-318, Leici, China) and the salinity meter (RHS-10ATC, Ade Advanced Optics, China). TOC and TN were measured using the Shimadzu TOC-L Total Organic Carbon Analyzer. The TOC-L analyzer adopts the 680 °C combustion catalytic oxidation method to determine the total carbon contents of samples. The NH_3-N content was determined with the nano-reagent-spectrophotometric method based on the absorbance at a wavelength of 420 nm. BOD is defined as the oxygen required for oxidizing the biodegradable organics in water or wastewater samples through a biochemical process. It is a measurement of the organic strength of the samples. Consequently, the BOD was obtained based on the change of DO in 5-day incubation. A Dionex ICS-2000 IC with an AD25 absorbance (230 nm) and a DS6 heated conductivity detector (35 °C) was used to measure the SO_4^{2-} and Cl^- levels. Finally, the Chl-a (mg/m^3) can be determined based on the absorbance.

Table S5. Physicochemical parameters of the seawater in the water tank every week

Time	pH	Temperature (°C)	DO (mg/L)	Cond (ms/cm)	TOC (mg/L)	TN (mg/L)	NH3-N (mg/L)	BOD (mg/L)	Salinity (‰)	SO_4^{2-} (g/L)	Cl^- (g/L)	Chl-a (mg/m^3)
2022/1/19-1 week	8.76	19.5	5.54	49.6	7.13	0.4627	2	0.8	35	2.68	27.7	3.4
2022/1/26-2 week	9.10	20.2	4.6	57.4	8.05	0.7232	2	1.4	37	2.61	25.4	3.6
2022/2/2-3 week	8.14	19.8	6.12	59.8	10.12	0.6279	1.8	1.1	35	2.57	26.8	5.4
2022/2/9-4 week	8.03	20.4	4.24	57.6	12.37	0.8941	1	0.8	42	2.62	27.5	4.2
2022/2/16-5 week	8.00	20.5	4.98	61.5	13.46	1.123	1.5	1.0	46	2.59	26.1	6.7
2022/1/23-6 week	8.12	21.2	5.32	63.2	15.58	2.405	1	1.2	45	2.64	27.2	6.5
Changing trend	/	/	/	+	+	+	/	/	+	/	/	+

During the corrosion experiment, the conductivity, TOC, TN, salinity, and *Chlorophyll* content increased because of the attachment and growth of bacteria and phytoplankton. When placing the concrete specimens in the running seawater, the leaching of calcium from calcium hydroxide would result in a decrease in surface pH [21]; meanwhile, the discharge of the pore solution from the concrete with high alkalinity would increase the pH

of the seawater in the tank [22] (Table S5). Thus, the pH of the seawater in the water tank was higher than the pH of the sampled seawater (Table S4). This increase in solution pH in the water tank would result in a small impact on the MIC on concrete according to the literature [22]. Similarly, the conductivity and salinity of the seawater in the tank also increased due to the leaching of calcium. As for the increase in TOC, TN, and *Chlorophyll* contents, these might be caused by the biofilm growth and the accumulation of microorganisms in a smaller water tank (Figure S4b).

Section S5. Measurements of surface sulfate concentrations and $CaCO_3$ contents

After the corrosion, the exposed surface of the concrete was washed using a high-pressure washer with 4000 mL of deionized water to remove biofilms that had formed with surface corrosion products. The wash-off water was homogenized using a magnetic mixer for 2 h before subsamples were placed in a sulfide antioxidant buffer solution [23]. The soluble sulfate was analyzed by ion chromatography and the surface sulfate concentration was calculated based on the soluble sulfate concentration and the area of the concrete surface.

Moreover, the $CaCO_3$ content in the concrete biofilms that had formed was measured using the acid pickling method [24]. After measuring the soluble sulfate, the wash-off water was filtered, and the insoluble products were initially dried and weighed, and then washed with 0.1 mol/L of HCl, followed by several rinses with deionized water. Finally, these samples were dried and re-weighed. The mass of the precipitated $CaCO_3$ was calculated as the dry weight loss and the $CaCO_3$ content was calculated based on the precipitated $CaCO_3$ mass, and the area of the concrete surface.

Section S6. Measurements of mass loss, AVPV, strength reduction, and sulfate concentrations in concrete

Before the experiment, the concrete specimens were first dried in an oven at 100±2 °C for 24 h, cooled to a temperature of 25±2°C, and weighed as the initial mass (M1). After the corrosion, the concrete specimens were drowned in deionized water for 48 h. The specimens were then dried and weighed again (M2) to obtain the mass loss (M1-M2). Then, the apparent volume of permeable voids (AVPV), an indicator of permeability, was obtained following the method described in the literature [25]. The specimens were immersed in deionized water at 25±2 °C for 48 h and boiled for a period of 5.5±0.5 h, after which they were kept in the deionized water until cooled to a final temperature of 25±2°C and weighted (M3). Finally, each specimen was suspended on a rack and immersed in deionized water at 25±2°C and the mass was recorded as M4. The AVPV was calculated according to **Eq. 1**.

$$AVPV = \frac{(M3-M1)}{M3-M4} \times 100\% \quad (1)$$

In addition to mass loss, compressive strength reduction is also a valuable indicator to study corrosion rates [26]. Compressive strength tests were conducted using a hydraulic press machine at a speed of 0.6 MPa/s, and the maximum obtained values were assumed to indicate the strength of the concrete specimens.

According to Fick's second law, the sulfate concentrations in concrete are related to corrosion depth and exposure time due to ion diffusion (**Eq. 2**) [27]. The transport of

bacteria in pores or cracks affected the concentration of ions in the concrete [28], resulting in different corrosion depths and diffusion coefficients. To determine sulfate concentrations at different depths in concrete, using a grinding machine the specimens were ground into powder at the top surface at 1 mm depth intervals up to a depth of 10 mm, and the interior was ground at 2 mm intervals along the length of the specimens up to a depth of 20 mm. For each depth interval, a powder sample weighing 25 g was collected and dried at 40 °C for 24 h, then cooled to room temperature. The sulfate concentrations at different depths were obtained using the barium sulfate gravimetric method [29]. Based on **Eq. 2**, the initial concentration of sulfate in concrete was considered to be zero, and the diffusion coefficient was calculated according to the sulfate concentrations at different depths.

$$\mathrm{C}(\mathrm{x},\mathrm{t}) = C_0 + (C_s - C_0)\left[1 - \mathrm{erf}\left(\frac{x-\Delta x}{2\sqrt{Dt}}\right)\right], \mathrm{erf}(\mathrm{x}) = \frac{2}{\sqrt{\pi}}\int_0^x e^{-\eta^2} d\eta \qquad (2)$$

where $\mathrm{C}(\mathrm{x},\mathrm{t})$ is the concentration of ions at a distance x from the exposed concrete surface at the exposure time t; C_0 represents the initial ion concentration (wt%); C_s is the ion concentration near the surface (wt%); D is the diffusion coefficient (m^2/s); and $\mathrm{erf}(\mathrm{x})$ represents the error function.

Section S7. qPCR procedure

As an external standard, a synthetic plasmid with 10^2-10^8 copies of the relevant gene per reaction was employed. In accordance with our previous study [30], the qPCR reaction was carried out in a 20-μL volume containing 10 μL of Power SYBR™ Green PCR Master Mix (Life Technologies), forward and reverse primers of the 16S rRNA gene (5′ → 3′: TCCTACGGGAGGCAGCAGT/GGACTACCAGGGTATCTAATCCTGTT) [31] at a final concentration of 250 nM for both primers, 1 μL of template DNA (concentration as low as 0.01ng/L), and a certain volume of UltraPure™ DNase/RNase-Free Distilled Water (Thermo Fisher Scientific). The amplification procedure included a 10 min at 95 °C, followed by 40 cycles of 15 s at 95 °C, annealing for 1 min at 60 °C, and extension for 45 s at 72 °C. In the end, a melt curve analysis was conducted to verify the specificity of the primer sets (the melt temperature of the amplicon at 84.2°C). All of the samples, standards, and negative controls were run in triplicate, with the efficiency of the amplification at around 97% and R^2 of 0.98.

The forward and reverse primers of *dsrB* were DSRp2060F (5′ → 3′: CAACATCGTYCAYACCCAGGG) and DSR4R (5′→3′: GTGTAGCAGTTACCGCA) [32, 33]. According to the method in the literature [17], the amplification program consisted of an initial denaturation step of 15 s at 95 °C, followed by 45 cycles of 10 s at 95 °C, annealing at 58 °C for 1 min, and extension for 30 s at 72 °C, with fluorescence measurement at the end of each extension. The amplification cycles were followed by a final extension for 3 min at 72 °C, and a melt curve analysis. For the melt curve

analysis, the amplicons were first denatured at 95 °C, followed by an annealing step at 60 °C and a progressive denaturation to 95 °C with a temperature increase of 2.2 °C/s and continuous fluorescence measurement. The efficiency of the amplification was around 98.5% and R^2 was 0.98.

Section S8. SRB communities

Table S6. Commonly studied SRB in marine corrosion

Phylum	Class	Genus_or_species
Aquificota	Aquificia	*Aquifex_pyrophilus*
Aquificota	Aquificia	Balnearium_lithotrophicum
Aquificota	Aquificia	Desulfurobacterium_crinifex
Aquificota	Aquificia	Desulfurobacterium_pacificum
Aquificota	Aquificia	Desulfurobacterium_thermolithotrophum
Aquificota	Aquificia	*Persephonella_guaimasensis*
Aquificota	Aquificia	Persephonella_marina
Aquificota	Aquificia	Thermocrinis_ruber
Aquificota	Aquificia	Thermosulfidibacter_takaii
Aquificota	Aquificia	*Thermovibrio_ammonificans*
Aquificota	Aquificia	Thermovibrio_guaymasensis
Aquificota	Aquificia	Thermovibrio_ruber
Bacillota	Clostridia	Ammonifex_degensii
Bacillota	Clostridia	Carboxydothermus_pertinax
Bacillota	Clostridia	Clostridium_sulfidigenes
Bacillota	Clostridia	Clostridium_thiosulfatireducens
Bacillota	Clostridia	Clostridium_tunisiense
Bacillota	Clostridia	Desulfitibacter_alkalitolerans
Bacillota	Clostridia	Desulfitispora_alkaliphila
Bacillota	Clostridia	Desulfitobacterium_chlororespirans
Bacillota	Clostridia	Desulfitobacterium_dehalogenans
Bacillota	Clostridia	Desulfitobacterium_hafniense
Bacillota	Clostridia	Desulfitobacterium_metallireducens
Bacillota	Clostridia	Desulfosporosinus_acididurans
Bacillota	Clostridia	Desulfosporosinus_acidiphilus
Bacillota	Clostridia	Desulfosporosinus_auripigmenti
Bacillota	Clostridia	Desulfosporosinus_meridiei
Bacillota	Clostridia	Desulfosporosinus_orientis
Bacillota	Clostridia	Desulfotomaculum_geothermicum
Bacillota	Clostridia	Desulfotomaculum_intricatum
Bacillota	Clostridia	Desulfotomaculum_reducens
Bacillota	Clostridia	Desulfotomaculum_salinum
Bacillota	Clostridia	Desulfotomaculum_thermosubterraneus
Bacillota	Clostridia	Dethiobacter_alkaliphilus
Bacillota	Clostridia	Ercella_succinogenes
Bacillota	Clostridia	Halanaerobium_congolense
Bacillota	Clostridia	Halarsenatibacter_silvermanii
Bacillota	Clostridia	Sporanaerobacter_acetigenes
Bacillota	Clostridia	Thermoanaerobacter_sulfurophilus

Bacteroidota	Bacteroidia	Petrimonas_sulfuriphila
Caldisericota	Caldisericia	Caldisericum_exile
Calditrichota	Calditrichae	Caldithrix_abyssi
Campylobacterota		Caminibacter
Campylobacterota		Hydrogenimonas
Campylobacterota		Lebetimonas
Campylobacterota		Nautilia
Campylobacterota		Nitratiruptor
Campylobacterota		Sulfurimonas
Campylobacterota		Sulfurospirillum
Campylobacterota		Sulfurovum
Campylobacterota		Thioreductor_incertae_sedis
Campylobacterota		Wolinella_succinogenes
Chrysiogenota	Chrysiogenetes	Desulfurispirillum_alkaliphilum
Coprothermobacterota	Coprothermobacteria	Coprothermobacter_proteoliticus
Deferribacterota	Deferribacteres	Deferribacter_desulfuricans
Deferribacterota	Deferribacteres	Geovibrio_thiophilus
Deinococcota	Deinococci	Oceanithermus_desulfurans
Pseudomonadota	Gammaproteobacteria	Acidithiobacillus_ferrooxidans
Pseudomonadota	Gammaproteobacteria	Pseudomonas_mendocina
Pseudomonadota	Gammaproteobacteria	Shewanella_putrefaciens
Spirochaetota	Spirochaetia	Spirochaeta_perfilievii
Spirochaetota	Spirochaetia	Spirochaeta_smaragdinae
Synergistota	Synergistia	*Anaerobaculum_mobile*
Synergistota	Synergistia	Anaerobaculum_thermoterrenum
Synergistota	Synergistia	Dethiosulfovibrio_acidaminovorans
Synergistota	Synergistia	Dethiosulfovibrio_marinus
Synergistota	Synergistia	Dethiosulfovibrio_peptidovorans
Synergistota	Synergistia	Dethiosulfovibrio_russensis
Synergistota	Synergistia	*Thermanaerovibrio_acidaminovorans*
Synergistota	Synergistia	Thermanaerovibrio_velox
Synergistota	Synergistia	Thermovirga_lienii
Thermodesulfobacteriota		Caldimicrobium_exile
Thermodesulfobacteriota		Desulfobacter_postgatei
Thermodesulfobacteriota		Desulfobacterium
Thermodesulfobacteriota		Desulfobotulus_alkaliphilus
Thermodesulfobacteriota		Desulfobulbus_propionicus
Thermodesulfobacteriota		Desulfomicrobium_baculatum
Thermodesulfobacteriota		Desulfomonile_tiedjei
Thermodesulfobacteriota		Desulfonatronovibrio_thiodismutans

Thermodesulfobacteriota		Desulfonatronum_thioautotrophicum
Thermodesulfobacteriota		Desulfovermiculus_halophilus
Thermodesulfobacteriota		Desulfovibrio
Thermodesulfobacteriota		Desulfurella
Thermodesulfobacteriota		Desulfurivibrio_alkaliphilus
Thermodesulfobacteriota		Desulfuromonas
Thermodesulfobacteriota		Desulfuromusa
Thermodesulfobacteriota		Geoalkalibacter_subterraneus
Thermodesulfobacteriota		Geobacter
Thermodesulfobacteriota		Hippea_maritima
Thermodesulfobacteriota		Pelobacter
Thermodesulfobacteriota		Thermodesulfobacterium_geofontis
Thermotogota	Thermotogae	Fervidobacterium_changbaicum
Thermotogota	Thermotogae	Fervidobacterium_islandicum
Thermotogota	Thermotogae	Fervidobacterium_nodosum
Thermotogota	Thermotogae	Fervidobacterium_riparium
Thermotogota	Thermotogae	Geotoga_petraea
Thermotogota	Thermotogae	Geotoga_subterranea
Thermotogota	Thermotogae	Marinitoga_camini
Thermotogota	Thermotogae	Marinitoga_hydrogenitolerans
Thermotogota	Thermotogae	Marinitoga_okinawensis
Thermotogota	Thermotogae	Marinitoga_piezophila
Thermotogota	Thermotogae	Mesotoga_infera
Thermotogota	Thermotogae	Mesotoga_prima
Thermotogota	Thermotogae	Petrotoga_mexicana
Thermotogota	Thermotogae	Petrotoga_miotherma
Thermotogota	Thermotogae	Petrotoga_mobilis
Thermotogota	Thermotogae	Thermosipho_aficanus
Thermotogota	Thermotogae	Thermotoga_lettingae
Thermotogota	Thermotogae	Thermotoga_maritima
Thermotogota	Thermotogae	Thermotoga_naphthophila
Thermotogota	Thermotogae	Thermotoga_neapolitana

Source: https://en.wikipedia.org/wiki/Sulfur-reducing_bacteria#Taxonomy

Section S9. SOB communities

Table S7. Commonly studied SOB in concrete corrosion

Phylum	Class	Genus_or_species	Source
Pseudomonadota	Acidithiobacillia	*Acidithiobacillus_ferrooxidans*	**https://en.wikipedia.org/wiki/Microbial_oxidation_of_sulfur#cite_note-71**
Chlorobi	Acidithiobacillia	*Acidithiobacillus_thiooxidans*	
Cyanobacteria	Cyanophyceae	*Calothrix_sp.*	
Cyanobacteria	Chlorobia	*Chlorobium_thiosulphatophilum*	
Proteobacteria	Gammaproteobacteria	*Chromatium_sp.*	
Proteobacteria	Gammaproteobacteria	*Chromatium_vinosum*	
Proteobacteria	Deltaproteobacteria	*Desulfobulbus_propionicus*	
Proteobacteria	Deltaproteobacteria	*Desulfocapsa_sulfoexigens*	
Proteobacteria	Deltaproteobacteria	*Desulfocapsa_thiozymogenes*	
Proteobacteria	Deltaproteobacteria	*Desulfovibrio_sulfodismutans*	
Proteobacteria	Gammaproteobacteria	*Ectothiorhodospira_shaposhnikovii*	
Proteobacteria		*Oscillatoria_sp.*	
Proteobacteria	Alphaproteobacteria	*Starkeya_novella*	
Proteobacteria	Epsilonproteobacteria	*Sulfurimonas_denitrificans*	
Proteobacteria	Gammaproteobacteria	*Thialkalivibrio_versutus*	
Proteobacteria	Betaproteobacteria	*Thiobacillus_denitrificans*	
Proteobacteria	Betaproteobacteria	*Thiobacilli_intermedius*	
Proteobacteria	Betaproteobacteria	*Thiobacilli_neopolitanus*	
Proteobacteria	Betaproteobacteria	*Thiobacilli_thiooxidans*	
Proteobacteria	Betaproteobacteria	*Thiobacillus_concretivorus*	
Proteobacteria	Betaproteobacteria	*Thiobacillus_denitrificans*	
Proteobacteria	Betaproteobacteria	*Thiomonas_intermedia*	
Proteobacteria	Betaproteobacteria	*Thiobacillus_intermedius*	
Pseudomonadota	Betaproteobacteria	*Thiobacillus_neopolitanus*	
Pseudomonadota	Betaproteobacteria	*Thiobacillus_thiooxidans*	
Pseudomonadota	Gammaproteobacteria	*Thiomicrospira_sp. CVO*	
Proteobacteria	Deltaproteobacteria	*Desulfovibrio_vulgaris*	[34]
Pseudomonadota	Gammaproteobacteria	*Halothiobacillus_neapolitanus*	[35]
Proteobacteria	Betaproteobacteria	*Thiobacilli_thioparus*	[36]
Proteobacteria	Betaproteobacteria	*Thiobacillus_ferrooxidans*	[35]
Proteobacteria	Betaproteobacteria	*Thiobacillus_neapolitanus*	[37]
Proteobacteria	Betaproteobacteria	*Thiobacillus_novellus*	[35]
Proteobacteria	Betaproteobacteria	*Thiobacillus_perometabolis*	[38]
Proteobacteria	Betaproteobacteria	*Thiobacillus_thioparus*	[35]
Proteobacteria	Betaproteobacteria	*Thiobacillus_tioparus*	[21]

Section S10. Summary of the results

Table S8. Summary of the results of the laboratory concrete corrosion experiment

Sample No.	Inoculation	Treatment	Properties of concrete		Biofilm		
			Sulfate in concrete	Surface sulfate, AVPV, mass loss, and strength reduction	Morphology characterizations	Bacterial communities	Corrosive bacterial communities
F-0	Submerged (flow velocity: 20 to 35 cm/s)	MIC	Sulfate concentrations, diffusion coefficients, and leaching depths:<br>F-0 > F-1 > F-2 > F-3<br>T-0 > T-1 > T-2 > T-3<br>CF-0 > F-3<br>CT-0 > T-3	• MIC > BCCI > CC<br>• T group > F group<br>• A higher UPB concentration resulted in a smaller AVPV, mass loss, and strength reduction	A thick and uneven biofilm with microbes embedded	• The difference in the bacterial communities between the MIC and BCCI samples and between the F and T groups gradually decreased	• BCCI samples had smaller total/relative abundances of SRB than MIC samples, especially for the sample with a higher UPB concentration<br>• The submerged group had much higher proportions of SRB<br>• There were no clear differences in SOB abundance between F and T groups and between the MIC and BCCI samples
F-1		BCCI ~1×10^6 CFU/mL			Uniform and compact biofilm with $CaCO_3$ crystals		
F-2		BCCI ~5×10^6 CFU/mL					
F-3		BCCI ~1×10^7 CFU/mL					
CF-0		CC			Less gypsum and ettringite than MIC		
T-0	Tidal (flow velocity: 20 to 35 cm/s)	MIC			Similar characteristics were observed, but several cracks were generated		
T-1		BCCI ~1×10^6 CFU/mL					
T-2		BCCI ~5×10^6 CFU/mL					

T-3		BCCI ~1×10^7 CFU/mL
CT-0		CC

For the submerged group, the corrosion resulted in a decrease in surface pH for all samples. After only 42 days of exposure, the decrease in the range of pH reached about 3.0 for concrete specimens with MIC (F-0), indicating a serious condition of corrosion [23], which was significantly larger than those specimens with CC (CF-0, $p < 0.01$). Due to several constraints (physicochemical parameters or bacterial composition), a variety of bacterial suspensions for biomineralization were proposed for various materials and environments [39-41]. UPB was commonly used to produce precipitates; thus, the UPB in seawater was specifically enriched in this study to inhibit corrosion. Eventually, the BCCI samples had smaller decreasing ranges of pH than F-0. The pH values varied with the UPB concentrations, generally following the order of F-3 > F-2 > F-1, indicating less production of sulfuric acid for F-3. Concerning the tidal groups, their ranges of decrease were much smaller than those of the submerged groups. Similarly, T-3 with the highest UPB concentration had the smallest range of decrease of surface pH.

Sulfate concentration can be used to quantitatively compare the ultimate generation of sulfuric acid that leads to corrosion [23]. The surface sulfate

concentration of F-0 was the highest. Comparably, the surface sulfate concentrations of F-3 and CF-0 were 43.3% and 56.6%, respectively, less than that of F-0, with a highly significant difference detected between F-0 and F-3, F-0 and CF-0 ($p < 0.01$) and a significant difference between F-3 and CF-0 ($p < 0.05$). The tidal groups had lower surface sulfate concentrations than the submerged groups due to a higher surface pH. Biomineralization resulted in a decrease in the production of sulfuric acid.

A previous study reported that microbial activity can spread across the entire zone of deterioration rather than just at the layers of corrosion close to the surface [42]. The microbes in the concrete deterioration zone accelerated their penetration directly into the concrete weakening the internal structure of the concrete [43]. The sulfate concentrations in the MIC samples (F-0 and T-0) were indeed much higher than those in the CC samples for both groups (**Figures 2b and c**); therefore, the MIC samples had larger diffusion coefficients and leaching depths (**Figure 2d**). The biomineralization effectively decreased the sulfate concentrations in the concrete, and the internal sulfate concentrations were lower for the samples with a higher UPB concentration (F-3 and T-3). This suggests that the biomineralized film acted as a protective layer controlling the diffusion of sulfate, leading to smaller diffusion coefficients and leaching depths for F-3 and T-3 than for the CC samples in the same group. In addition to the aggressive ion attack, the tidal groups also experienced alternating wetting-drying processes. High concentrations of sulfate can be transported

from the surface to the inner layers of the concrete by diffusion and penetration due to changes in the pressure gradient at the pores and cracks during the wetting-drying process [44]. As a result, the internal sulfate concentrations in the tidal groups were higher than the ones in the submerged groups despite fewer surface corrosion products. The higher internal sulfate levels also contributed to larger diffusion coefficients and greater leaching depths.

The presence of sulfate in a chloride environment increased porosity, accelerating the corrosion of mortar [45]. In this study, the corrosion indeed resulted in several small holes on the surface of the concrete (Figure S3) and the AVPV increased from 10.3 in the original state (S-0) to 12.5 and 10.8 in F-0 and T-0, respectively, after corrosion (**Figure 2e**). The tidal inoculation resulted in a larger increase in AVPV than in the submerged inoculation because of the dual impact of microbes and the wetting-drying process. Furthermore, the BCCI samples had relatively smaller AVPV than the MIC samples, despite different inoculations. The T-3 group had a significantly smaller AVPV than the T-2 group ($p < 0.05$), which was mainly attributed to a higher concentration of UPB. The more compact biomineralized film might have mitigated the generation of small holes or the UPB might have healed the surface cracks or pores [28], both of which could lead to a decrease in AVPV.

Mass loss and strength reduction are key factors in determining the degree of corrosion [46]. **Figure 2f** shows that the mass loss decreased in the concrete, with MIC > BCCI > CC. The results were comparable to previously reported mass loss in laboratory studies [25]. F-0 and T-0 showed significantly higher ($p < 0.05$) mass loss than F-3 and T-3. A highly significant difference ($p < 0.01$) was detected between F-0 and CF-0, and between T-0 and CT-0. From **Figure 2g**, compared with submerged groups, there was a larger reduction in the strength of the concrete in the tidal groups. The corrosion was inhibited by biomineralization, and the concrete with a high concentration of UPB was more corrosion-resistant, leading to a smaller reduction in strength. Some SRB communities can also induce the formation of $CaCO_3$ precipitation [47]; thus, we observed a small amount of $CaCO_3$ in F-0 and T-0 (**Figure 2h**). The enriched UPB in biomineralization utilized CO_2 to form $CaCO_3$ precipitation on the concrete surfaces; thus, about 10 times the $CaCO_3$ content was detected in F-3 and T-3 compared to F-0 and T-0, contributing to carbon neutrality.

Section S11. Physicochemical parameters and genotypic data analysis of seawater during corrosion

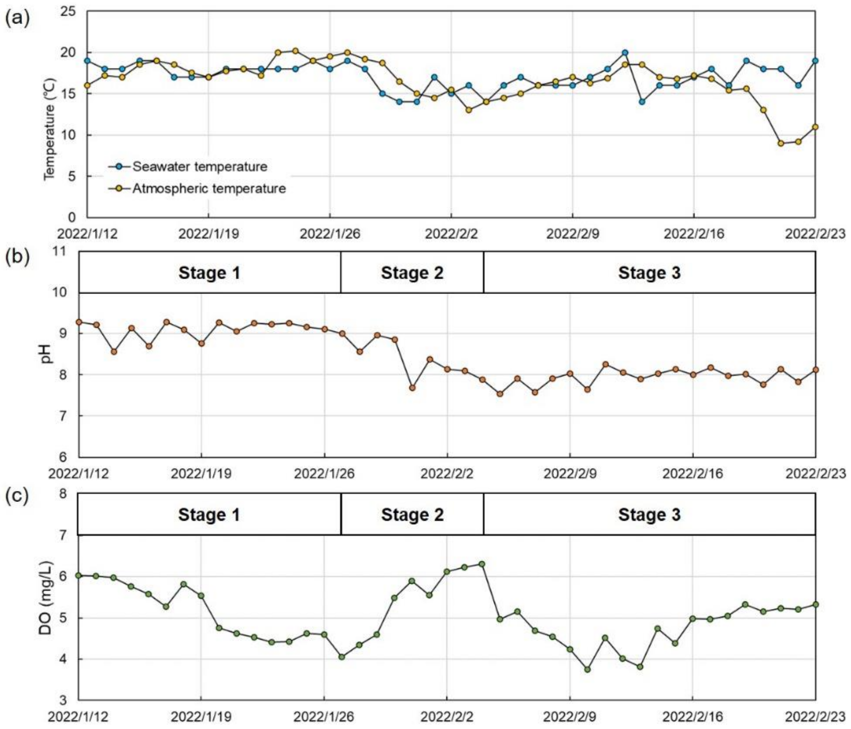

Figure S2. The evolution during the experiment for (a) the temperature of seawater in the water tank and atmospheric temperature; (b) the pH and (c) the DO of the seawater in the water tank.

Temperature is an important factor in corrosion in marine environments [27]. The atmospheric temperature was similar to the seawater temperature in the laboratory environment (Figure S2a). Therefore, the influence of temperature on the two groups (submerged group and tidal group) can be recognized as the same. Based on the changing pattern, the evolution of pH and DO of the seawater in the water tank were divided into three stages (Figure S2b and c). MIC for concrete is mainly related to the production of sulfuric acid. Most sulfate reducers prefer low-grade carbon sources such

as volatile fatty acids. A small fraction of them has evolved to possess the ability to use hexoses such as glucose as a carbon and energy source [48]. Some SRB species have developed the ability to use O_2 as an electron acceptor to oxidize an organic carbon [49]; so, the attachment and growth of bacteria resulted in the decrease of DO in the first stage [50]. With the corrosion evolution, several small holes can be seen on the concrete surface after only 14 days, especially for the submerged group (Figure S3). The highly alkaline environment inside the concrete neutralized the decrease of pH resulting from corrosion. Consequently, the pH of seawater in the water tank was still over 9.0, which was relatively higher than the pH of the seawater sampled (Table S5).

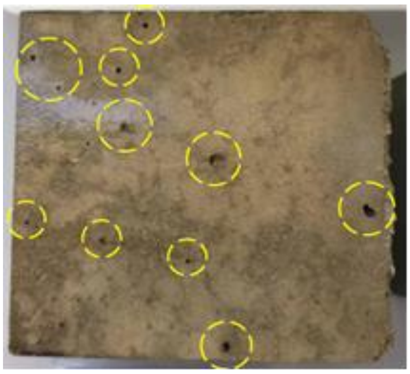

Figure S3. The concrete surface of F-0 after 14-day corrosion

In the second stage, the pH decreased to about 8.0, and the DO rapidly increased. In the third stage, the pH entered a fluctuation stage; DO first decreased and increased again. Moreover, after 14 days, the change on the surface of concrete specimens in the submerged group can be clearly observed (**Figure 1b**). The authors hypothesize there should be a combination effect of phytoplankton (*e.g.,* diatom and algae) and bacteria in stage 2 and stage 3 because the growth of phytoplankton would increase the DO [51]. On the 33rd day, the concentration of phytoplankton in the seawater in the water tank

was obtained, reaching 1.5×10^7 cells/L, which was much higher than the concentration of phytoplankton in Victoria harbor (4×10^6 cells/L, the data from Hong Kong Marine - Annual Marine Water Quality Reports). A control sample without concrete was prepared for comparison, and the concentration of phytoplankton was 6×10^6 cells/L. It indicated that the colonization of phytoplankton on the concrete surface accelerated the growth of phytoplankton in the seawater of the water tank.

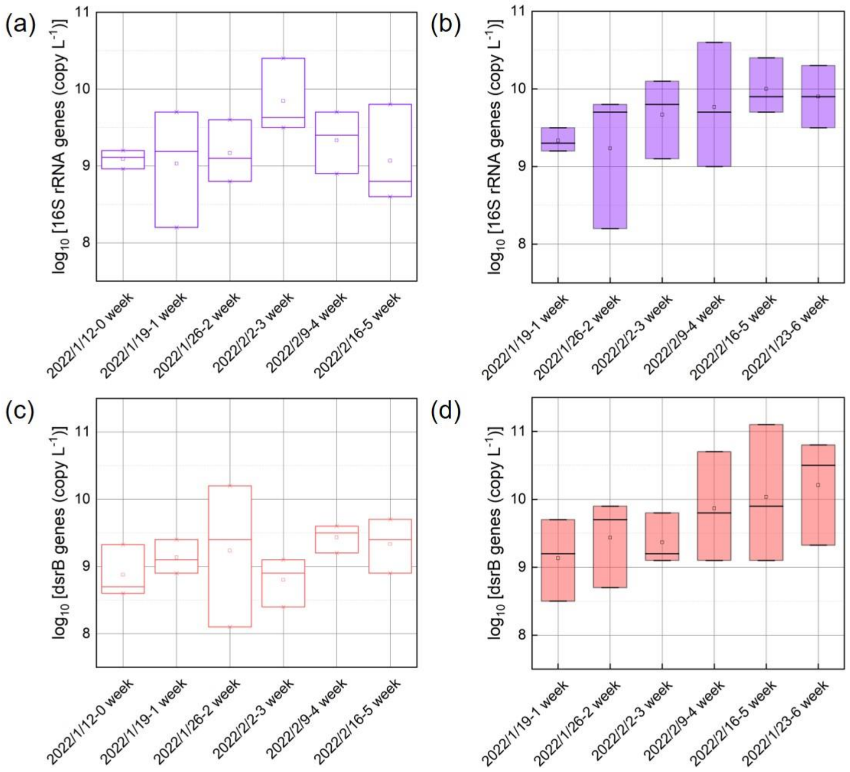

Figure S4. qPCR analysis for concentrations of (a) the 16S rRNA gene and (c) *dsrB* in the seawater sampled from the Tsim Sha Tsui Pier, and concentrations of (b) the 16S rRNA gene and (d) *dsrB* in the seawater in the water tank.

The DNA extraction and qPCR analysis were conducted for the sampled seawater and the seawater in the water tank every week. **Figure S4** shows the concentrations of both the 16S rRNA gene and *dsrB*. In general, the concentrations of the 16S rRNA and *dsrB*

genes were relatively consistent during the whole experimental process in the seawater sampled from the pier. Nevertheless, different from the less fluctuation in the sampled seawater, the 16S rRNA and *dsrB* concentrations of the seawater in the water tank both recorded an increase in the latter stage of the experiment, which might be due to the shifting from the formed concrete biofilms.

Section S12. Mechanical properties, morphology characterizations, and corrosion products

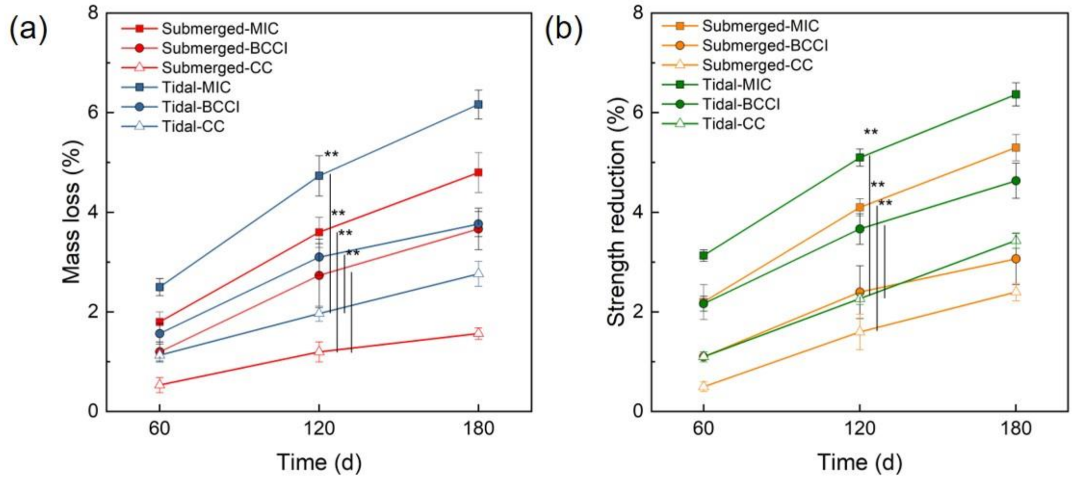

Figure S5. After the six-month corrosion, the change in (a) concrete mass and (b) compressive strength, respectively. The BCCI samples had the same biomineralization treatment condition as the F-3 and T-3 in Figure 2.

We conducted another corrosion experiment for six months, which was different from this current study and mainly focused on biofilm communities. The seawater conditions were different from the present study because of the different sampling seasons. However, the mass loss and strength reduction of the concrete samples were consistent with the results in the 42-day corrosion study. During the six-month corrosion, the initial mass loss ratio of MIC samples in the first two months was also about 1.8-2.5%, and 2-3% for the strength reduction ratio (Figure S5). After two months, however, the reduction rate became smaller. The mass loss ratio of MIC samples reached about 4-6% after six months, and 5-6% for the strength reduction ratio. The changing trends can also be observed in some previous studies on sulfate attacks [52-54]. Compared with MIC samples, the BCCI samples always had better resistance performance in the six-month study. Moreover, in our study, corrosion happened on every concrete surface,

which contributed to a relatively higher loss in 42 days. In addition, the concrete samples had much higher corrosion rates than real marine concrete infrastructure, because the degradation is directly proportional to the exposure area/volume ratio [55], which also contributed to the 2-3% loss in 42 days. Similar higher corrosion rates have been reported in several previous studies [54, 56, 57].

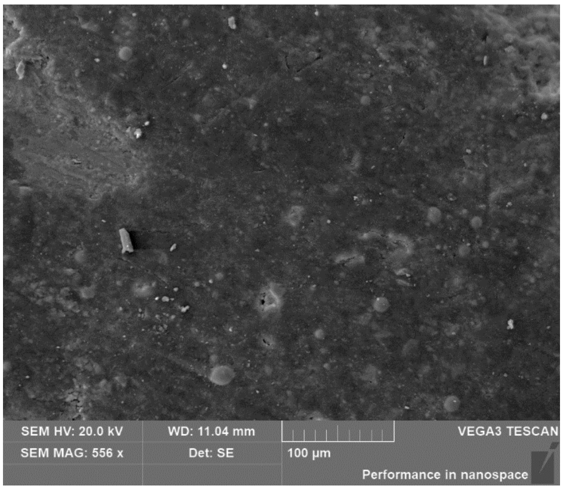

Figure S6. The SEM image of concrete without corrosion

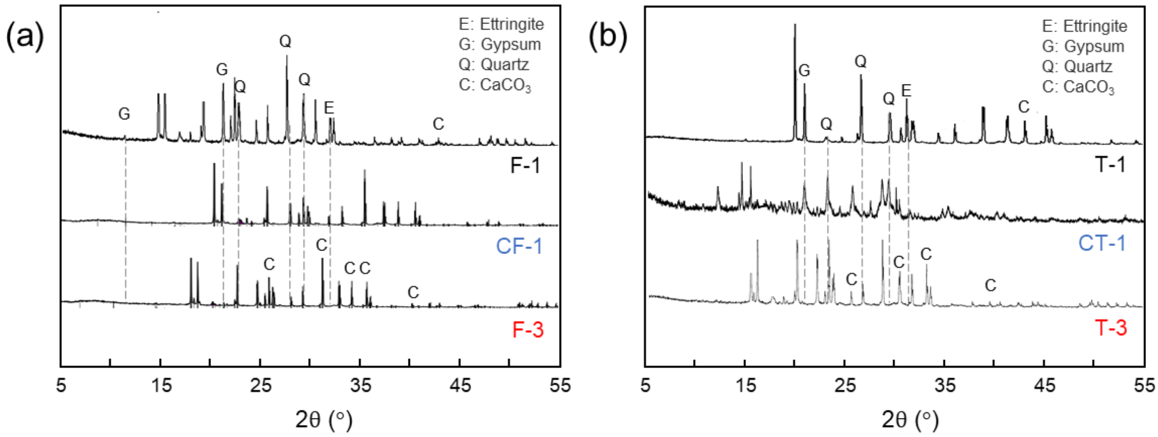

Figure S7. The XRD results of corrosion products: (a) the submerged groups; (b) the tidal groups.

Furthermore, the microscopic characteristics and mass percentages of different elements of the concrete biofilms were observed using SEM-EDX (TESCAN VEGA3, TESCAN, Czech Republic) [58, 59]. This equipment was operated at ~0.001 Pa and in secondary electron mode at a voltage of 20 kV, a beam current of 60 μA, and a working distance of ~10 mm. X-ray Diffraction (XRD) was used to identify the corrosion products [60]. The XRD (Rigaku SmartLab 9 kW-Advance) measurement was carried out in a 2θ range of 5° to 36° with a step width of 0.02°, and the scan speed was fixed at 30°/min. The XRD results can be seen in Figure S7. Gypsum and ettringite have formed in the MIC samples (F-0 and T-0) and the CC samples (CF-0 and CT-0). A large amount of $CaCO_3$ was determined in the BCCI samples (F-3 and T-3).

Section S13. qPCR results of biofilm on concrete surface

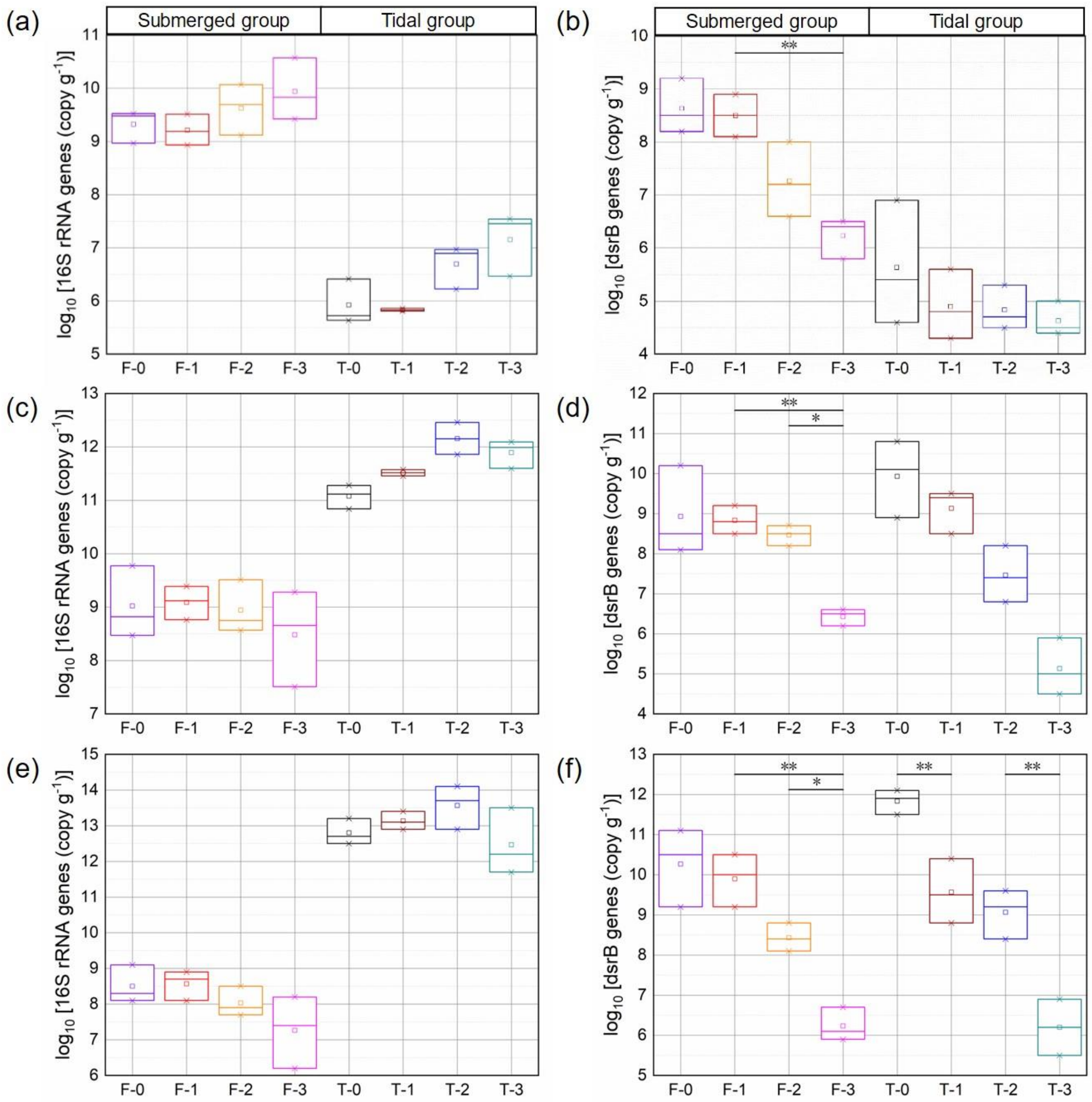

Figure S8. Evolution of concentrations of specific gene contents in concrete biofilms quantified by qPCR: (a) 16S rRNA gene after 14 days; (b) *dsrB* after 14 days; (c) 16S rRNA gene after 28 days; (d) *dsrB* after 28 days; (e) 16S rRNA gene after 42 days; (f) *dsrB* after 42 days.

After 14 days, the submerged groups had much higher concentrations of the 16S rRNA gene than the tidal groups (Figure S8a). For both two types of groups, a higher concentration of UPB

resulted in a higher concentration of the 16S rRNA gene (F-3 and T-3), but there is no significant difference ($p > 0.05$). The general patterns changed after 28 days. The concentrations of the 16S rRNA gene in the biofilm of tidal groups exceeded the submerged groups (Figure S8c) because the subsequent nonbacterial attachment in the tidal groups decreased the bacterial contents in the microbial community. Moreover, the F-3 and T-3 groups had relatively lower bacterial concentrations than the F-2 and T-2 groups, respectively. The reason might be that the pre-added UPB accelerated the formation of biofilm and the subsequent attachment of nonbacteria, which decreased the concentrations of the 16S rRNA gene in the F-3 and T-3 groups. The comparison results were similar after 42 days, while the difference between the submerged groups and the tidal groups became larger (Figure S8e). The differences between F-2 and F-3, and T-2 and T-3 also became larger; nevertheless, the differences were not significant ($p > 0.05$).

After 14 days, the submerged groups had much higher *dsrB* concentrations than the tidal groups (Figure S8b), which indicated that the submerged inoculation caused more severe microbial corrosion than the tidal inoculation. The covering of the rebar surface by corrosion products can limit SRB from accepting the electrons needed for their metabolic process [61]. In this study, the covering of the hybrid film also limited SRB, indicated by the lower *dsrB* concentrations in samples with biomineralization. The *dsrB* concentrations in biofilms of F-1 slightly decreased compared with F-0 and continued to decrease with increased UPB concentrations. Compared with the F-1, the *dsrB* concentrations of F-3 highly significantly decreased. The inhibiting effect for the tidal groups was much smaller than the submerged groups; however,

after 28 days, the biomineralization still resulted in larger differences among samples in the tidal group despite no significant differences ($p > 0.05$) (Figure S8d). For the submerged groups, the *dsrB* concentrations of F-3 significantly decreased compared with F-2 ($p < 0.05$) and were highly significantly lower than F-1 ($p < 0.01$). The comparison results were similar for the submerged groups after 42 days (Figure S8f). However, the comparison results were quite different for the tidal groups. The T-1 had highly significantly lower *dsrB* concentrations than the T-0 ($p < 0.01$). The *dsrB* concentrations highly significantly decreased again from T-2 to T-3 ($p < 0.01$).

For the submerged groups, the concentrations of the 16S rRNA gene gradually decreased due to the subsequent nonbacterial attachment and the decreasing rates were larger for the groups with higher concentrations of UPB (Figure 5a). For both the F-2 and F-3 groups, the concentrations of the 16S rRNA gene significantly decreased from the 14th day to the 42nd day ($p < 0.05$). Nevertheless, the concentrations of the 16S rRNA genes increased highly significantly from the 14th day to the 28th day for all the tidal groups ($p < 0.01$). For the T-0 and T-1, the concentrations of the 16S rRNA gene continued to increase significantly ($p < 0.01$) from the 28th day to the 42nd day. The biomineralization inhibited the growth of SRB; therefore, the increasing rate was smaller for the T-2 and T-3, especially the T-3 with no significant increase. The *dsrB* concentrations gradually increased for the F-0 and F-1 (Figure 5b), suggesting that a lower concentration of UPB did not exhibit a good corrosion inhibition effect. There was a significant increase from the 14th day to the 42nd day for F-1 ($p < 0.05$). For the F-2, the *dsrB* concentrations significantly increased from the 14th day to the 28th day

($p < 0.05$), while the data slightly decreased after 28 days because the formation of the mature biomineralized film limited the growth of SRB. For the F-3, the *dsrB* concentrations also decreased from the 28th day to the 42nd day. For the tidal groups, the *dsrB* concentrations always increased from the 14th day to the 42nd day. There were highly significant differences between the 14th-day data and the 28th-day data for the T-0, T-1, and T-2 ($p < 0.01$). However, the increasing rate became considerably smaller after 28 days. For the T-3, there was only a notable increase from the 14th day to the 42nd day, and their increase rate was much smaller than the other three tidal groups. The results show that the biomineralization method enabled a significant decrease in the numbers of SRB but did not affect the subsequent colonization of phytoplankton (Figure S9).

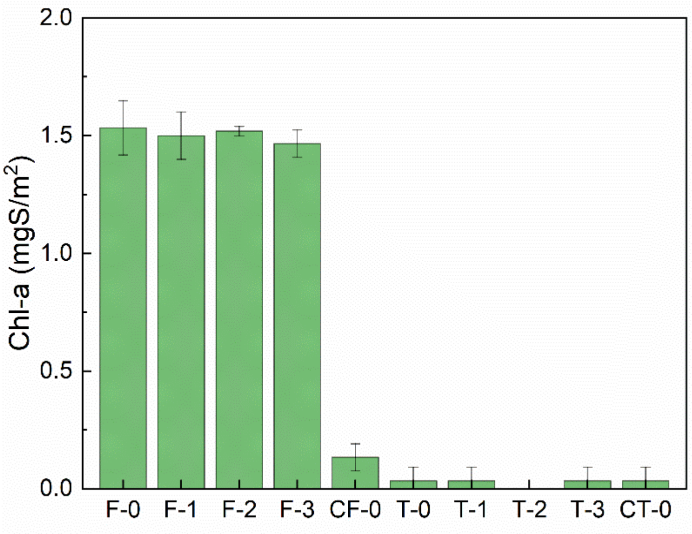

Figure S9. Comparison of Chl-a concentration for different concrete specimens after corrosion.

Section S14. Genotypic data analysis of biofilms

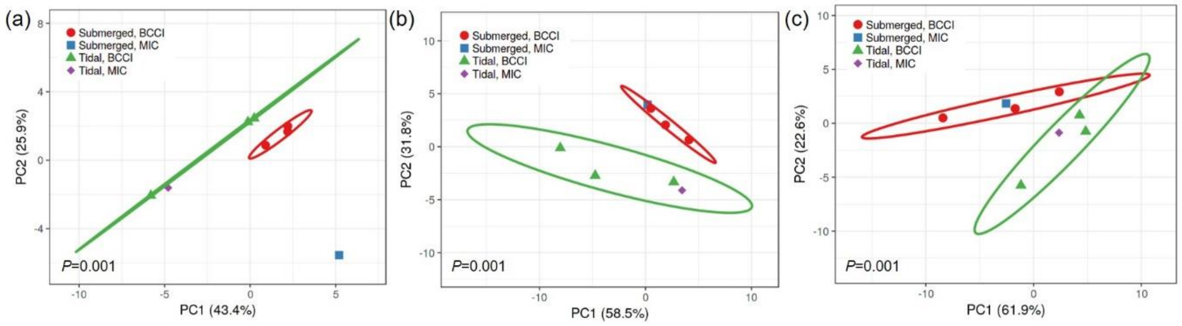

Figure S10. Microbial community differences as visualized with PCoA on (a) the 14th day, (b) the 28th day, and (c) the 42nd day. The permutational multivariate analysis of variance (PERMANOVA) was used for tests of inter-group differences. $P < 0.05$ was regarded as the criterion for statistical significance of the difference. Ovals indicate the 95% confidence intervals for each sample type.

The cluster of microorganisms was clearly separated among samples in the two different groups and there was also a large difference in the bacterial communities between the MIC and BCCI samples on the 14th day. However, the difference in the bacterial communities between the MIC and BCCI samples gradually decreased, as well as the difference between submerged and tidal groups. It indicated the formation of the biomineralized film resulted in different initial bacterial communities; however, subsequent similar communities colonized on top of the biomineralized film, eventually leading to increasingly similar community structures.

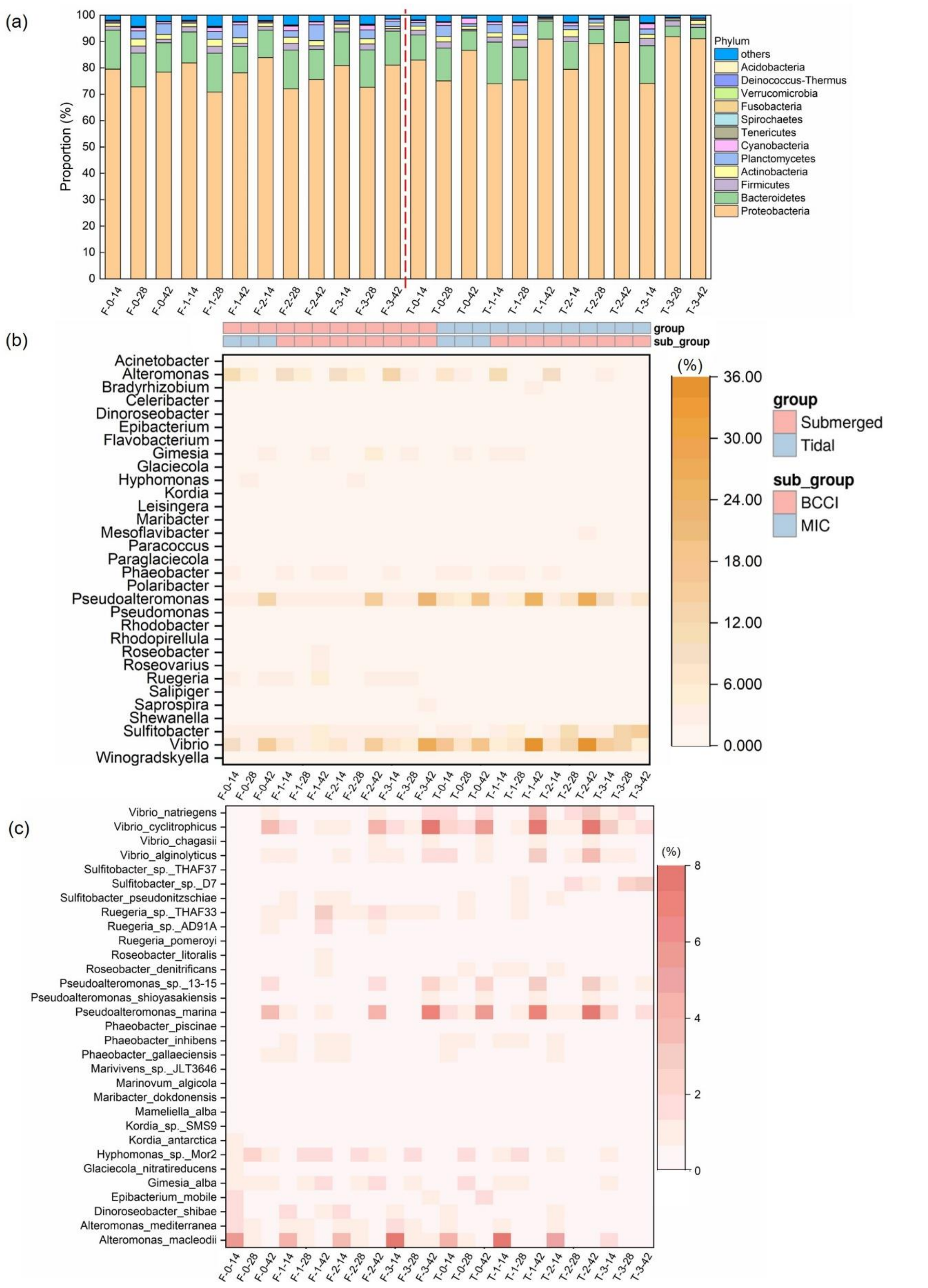

Figure S11. Dominant bacterial composition in concrete biofilms: (a) top 10 at the phylum level, (b) top 20 at the genus level, and (c) top 20 at the species level.

The bacterial communities in concrete biofilms were numerically dominated by *Proteobacteria* and *Bacteroidetes*, with respective average relative abundances of 77.3% and 12.6% for the submerged groups and 83.4% and 9.3% for the tidal groups (Figure S11a). The family *Rhodobacteraceae* was highly abundant in all of the concrete biofilms. There was a larger proportion of the genus *Sulfitobacter*, which was related to sulfur [62] from the family *Rhodobacteraceae*, and it was identified as one of the top 20 genera (Figure S11b). The members of this family, such as the chemoorganotrophic bacterium *Sulfitobacter sp.THAF37*, *Sulfitobacter sp.D7*, and *Sulfitobacter pseudonitzschiae*, were identified as being among the top 20 species (Figure S11c). The proportion of *Sulfitobacter sp.D7* for the submerged groups was smaller than that for the tidal groups. The proportion of *Sulfitobacter sp.THAF37* slightly changed with corrosion and there was a small difference between the submerged groups and the tidal groups. As for *Sulfitobacter pseudonitzschiae*, no clear pattern was observed in this study.

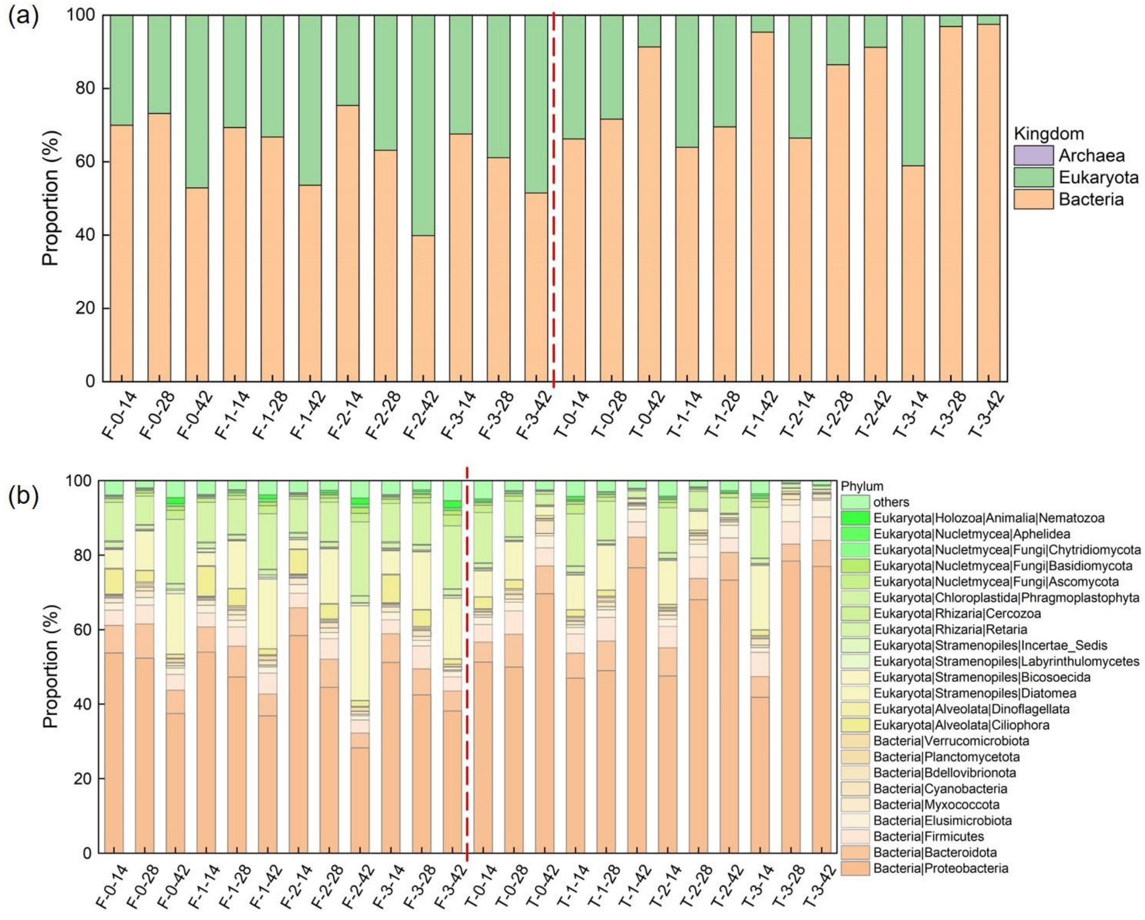

Figure S12. Comparison of biofilm profile during the corrosion experiment at the (a) kingdom and (b) phylum levels.

In general, the surface change brought about by bacterial attachment makes it easier for later colonizers to adhere, and the number of eukaryotes will rise on any surface exposed to the aquatic environment [63]. In this study, more eukaryotic taxa subsequently colonized on the surface of concrete in the submerged groups (Figure S12b).

Some of the field studies confirmed that *Acidithiobacillus* species are acidophilic SOB that dominate the microbial community (>50%) [64], while other studies showed an extremely low abundance of *Acidithiobacillus* species, suggesting that they may not be as important as people

have believed [65]. This study also found low proportions of the *Acidithiobacillus* species due to abundant bacterial species in the marine concrete biofilms. Although there were relatively larger proportions of *Acidithiobacillus* species in the submerged groups than in the tidal groups, the much higher concentrations of total bacteria in the tidal groups on the 28th and 42nd day finally resulted in higher absolute abundances of *Acidithiobacillus* species in the tidal groups because the tidal interface promoted the production of $H_2S(g)$ [66]. However, the low relative abundances of SRB in the tidal groups eventually still contributed to fewer corrosion products and lower concentrations of surface sulfate (**Figure 2a**).

In addition, acidophilic SOB such as *Acidithiobacillus thiooxidans* contained the same phospholipids as phosphatidyl glycerol; however, neutrophilic SOB such as *Starkeya novella* had more types and different compositions of phospholipids, indicating a potential diversity in function between them [67]. Two major different pathways for thiosulfate oxidation were confirmed between the two types of SOB [35, 68]. The difference in the diversity in function between the neutrophilic SOB and the acidophilic SOB in concrete biofilms may be the main reason for the different corrosion rates in different MIC environments (seawater, sewage structures, and wastewater treatment plants). As for the species *Sulfitobacter sp.THAF37*, *Sulfitobacter sp.D7*, and *Sulfitobacter pseudonitzschiae* in Figure S11c, there were no definitive studies to prove how they participated in the sulfur cycles in MIC; thus, they were not included in the SOB profiles despite high abundances.

Section S15. Different corrosion rates in different corrosion experiments

Table S9. Comparison of corrosion rates in different experiments

	Sample type	Sample size	W/C	Mass loss (%)	Strength reduction (%)	Mass loss rate (%/year)	Strength reduction rate (%/year)	Period	Reference	Immersed solution
1	SSC	100mm×100 mm×100 mm	0.38	1.90	2.23	16.51	19.42	42 days	Current study	Submerged in seawater
				2.40	3.35	20.86	29.13			Tidal in seawater
Acid corrosion (AC)										
1	OPCC	Φ100mm×200mm	0.7	2.50	5.00	6.33	12.65	144 days	61	1% sulfuric acid solution
				4.00	30.00	12.58	94.38	116 days		3% sulfuric acid solution
	AAC			3.50	0.00	8.86	0.00	144 days		1% sulfuric acid solution
				15.00	22.50	47.19	70.79	116 days		3% sulfuric acid solution
2	OPCC	100mm×100 mm×100 mm	0.6	2.50	33.33	10.00	133.33	3 months	69	2% sulfuric acid solution
	BAGC			0.70	6.90	2.80	27.59			
	OPCC			5.00	37.04	10.00	74.07	6 months		
	BAGC			2.50	8.62	5.00	17.24			
	OPCC			7.50	62.96	7.50	62.96	12 months		
	BAGC			3.50	31.03	3.50	31.03			
	OPCC			18.00	66.67	12.00	44.44	18 months		
	BAGC			10.00	34.48	6.67	22.99			
3	Concrete with CEM II	100mm×100 mm×100 mm	0.3-0.62	3.6-6.3	19.2-36.6	46.9-82.125	250.28-477.1	28 days	70	5% sulfuric acid solution
				9-12.3	38.6-57.2	58.66-80.17	251.59-372.82	56 days		
	Concrete with CEM		0.29-0.63	3.6-6.7	22.6-46.1	46.9-87.4	294.6-600.946	28 days		

	Ⅲ			8.6-12.6	43.3-62.7	56.05-82.125	288.22-408.67	56 days		
4	OPCC	100mm×100 mm×100 mm	0.34	20.00	38.00	115.00	218.50	140 days	[71]	pH1.1 sulfuric acid solution
	AAC			13.00	15.00	74.75	86.25			
	OPCC			7.00	23.00	40.25	132.25			pH1.6 sulfuric acid solution
	AAC			3.00	9.00	17.25	51.75			
	OPCC			4.00	18.00	23.00	103.50			pH2.0 sulfuric acid solution
	AAC			2.00	4.00	11.50	23.00			
5	GPM	50mm×50m m×50mm	0.5	23-39	30-52	99.9-169.46	130.357-225.95	12 weeks	[72]	5% sulfuric acid solution
6	OPCC	Φ40mm×40 mm	0.35	(-4)-3		(-48)-36		1 month	[73]	0.2 M sulphuric acid with pH 0.9
				1-10		6-60		2 months		
				7-13		28-52		3 months		
				13-20		39-60		4 months		
				17-22		40.8-52.8		5 months		
				24-30		48-60		6 months		
				30-38		51.4-65.14		7 months		
				34-43		51-64.5		8 months		
				38-48		50.6-64		9 months		
				44-52		52.8-62.4		10 months		
				47-56		51.27-61.09		11 months		
				51-62		51-62		12 months		
Acid corrosion + bacteria										
1	OPCC	20mm×20m m×10 mm	0.5	2.1-4.8		26.13-64.2		28days	[74]	pH 2 Bacterial medium of A. thiooxidans
		20mm×20m m×20 mm		1.1-4.8		6.6-28.8		2 months		
				4.6-17		18.4-68		3 months		
				6.3-18.2		12.6-36.4		6 months		
2	OPCC	20mm×20m	0.4	7.9		72.09		40 days	[75]	H_2S atmosphere for 2

	Polymer-modified concrete	m×50mm	0.34	5.9		53.84				days and 100 mg/l $(NH_4)_2SO_4$ and 10 mg/l K_2HPO_4) and Thiobacillus-like bacteria
			0.35	6.8		62.05				
			0.34	8.1		73.91				
			0.41	6.8		62.05				
3	OPCC	20mm×20mm×20 mm	0.34	6.25	1.67	228.13	60.83	10 days	76	pH2.0 sulfuric acid solution and A. thiooxidans
				7.50	5.00	136.88	91.25	20 days		
				18.75	16.67	228.13	202.78	30 days		
				21.88	43.33	199.61	395.42	40 days		
				25.00	53.33	182.50	389.33	50 days		
				31.25	70.00	190.10	425.83	60 days		
				37.50	81.67	195.54	425.83	70 days		
				40.63	83.33	185.35	380.21	80 days		
				43.00	85.00	174.39	344.72	90 days		
4	OPCC	20mm×20mm×50mm		9–11		64.4-78.7		51 days	37	4% Na_2S, 1.5 N HCl and T. neapolitanus T. *Thiooxidans T. ntermedius/T. novellus*
5	Concrete	18mm×18mm×20mm		5.8		7.84		270 days	77	10 ppm H_2S (g) with Thiobacillus neapolitarius, *T. intermedius, and T. thiooxidans*
Microbially induced corrosion (MIC)										
1	OPCC	50mm×50mm×20mm	0.38	4.65		4.98		335 days	25	Flowing river water
				4.04		4.32				Statistic river water
2	OPCC	40mm×18mm×8mm	0.4	35		35		365 days	78	Sewer atmosphere in field site
3	OPCC	Φ100mm×200mm	0.4		3.00		0.43	7 years	79	Marine tidal zone of the Gulf of Thailand
			0.45		5.00		0.71			

			0.5	19.00	2.71			
4	OPCC		0.38	1.82	23.70	28 days	56	Submerged in Persian Gulf seawater
				5.45	71.10			Tidal in Persian Gulf seawater
	OPCC with SF and FA			0.85-2.04	11.08-26.60			Submerged in Persian Gulf seawater
				1.02-5.08	13.30-66.28			Tidal in Persian Gulf seawater
5	HVFACP	20mm×20mm×20 mm	0.25	6.0-8.0	8.0-10.63	9 months	54	Tap water
				16.5-22.5	22-30			Submerged in seawater
				26-32	34.67-42.67			Tidal in seawater
6	AAS pastes	20mm×20mm×20 mm		12.00	36.00	3 months	57	Submerged in seawater
				25.00	50.00	6 months		
				37.00	37.00	12 months		
				16.00	64.00	3 months		Tidal in seawater
				37.00	74.00	6 months		
				49.00	49.00	12 months		
	AAS pastes with MK			11.0-15	33.0-45	3 months		Submerged in seawater
				21.0-30	42.0-60	6 months		
				34.5-39	34.5-39	12 months		
				20.0-21	80.0-84	3 months		Tidal in seawater
				31.0-40	62.0-80	6 months		
				49.0-53.0	49.0-53.0	12 months		

Note: Seawater sea sand concrete (SSC), ordinary Portland cement concrete (OPCC), alkali-activated concrete (AAC), blended ash geopolymer concrete (BAGC), glass power mortars (GPM), Acidithiobacillus ferrooxidans (T.f. bacterium), silica fume (SF), fly ash (FA), high-volume fly ash cement pastes (HVFACP), alkali-activated slag (AAS), microsize metakaolin (MK).

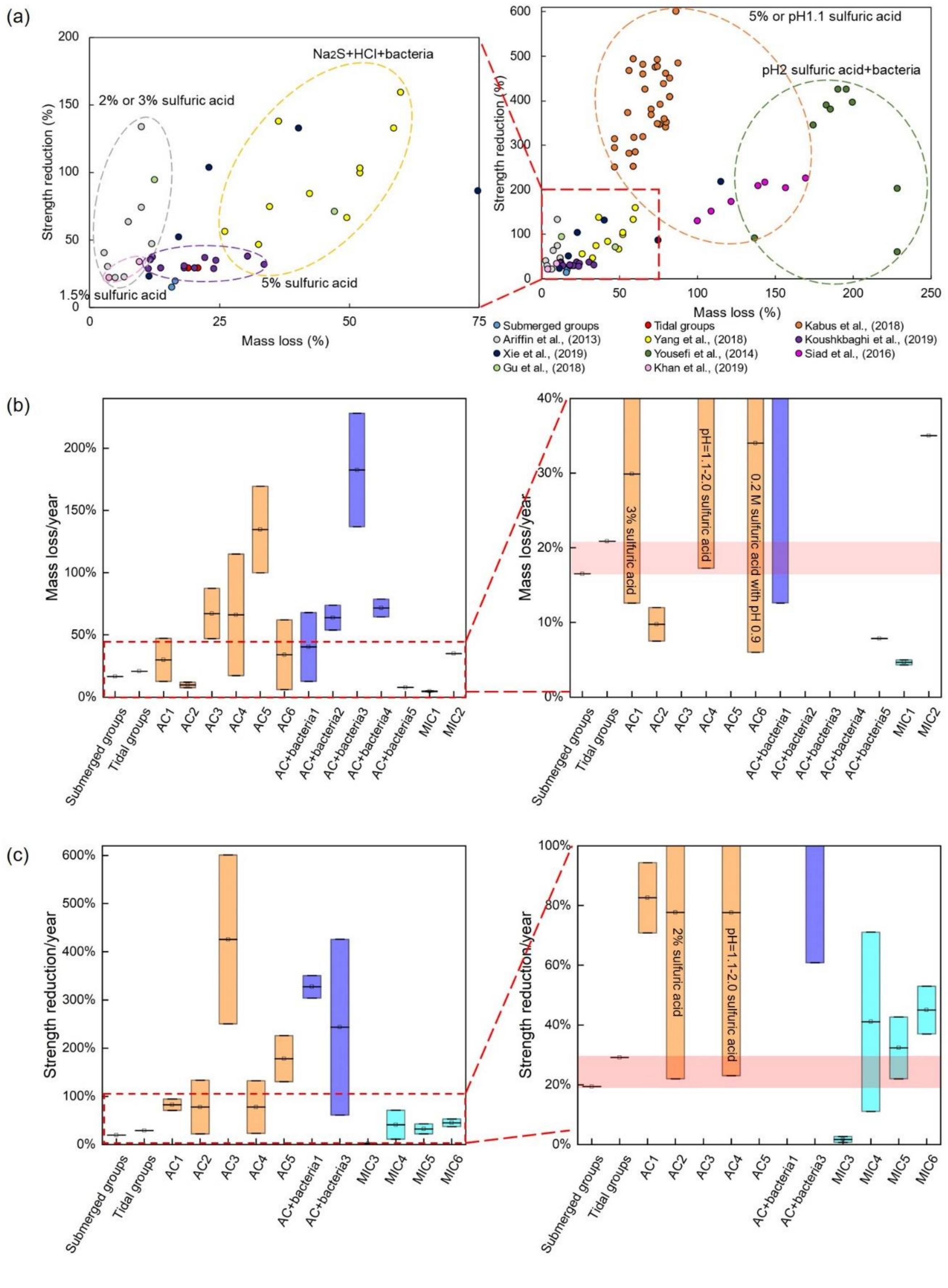

Figure S13. Comparison of corrosion rate between the current study and previous studies: (a) based on both the mass loss and strength reduction; (b) only based on mass loss; and (c) only based on strength reduction.

The data (mass loss and strength reduction) from several previous studies were converted into mass loss and strength reduction per year for convenient comparison. Researchers in civil engineering often used acid corrosion experiments as accelerated experiments to study the durability of concrete. Additionally, they did a chemical corrosion experiment using artificial seawater solution or other salt solutions. The researchers in environmental engineering did some studies on the influence of bacteria on corrosion by adding bacterial strains to the acid solutions. There were fewer studies on MIC corrosion, some of them using river water or seawater, or directly putting concrete specimens in marine environments. Concerning the corrosion rate evaluation, not all of the studies included both mass loss and strength reduction. Some studies only had data on mass loss or strength reduction. Therefore, the data from previous studies were divided into three cases and they were compared separately, as shown in Figure S13.

The comparison based on the mass loss and strength reduction reported that the corrosion rates in sulfuric acid solutions (5% or pH=1.1) or the solution with pH=2 sulfuric acid and bacteria were much higher (Figure S13a). The corrosion rates in this study were higher than in the 1.1% sulfuric acid solution in [80], even higher than some samples in the 2% sulfuric acid solution in [69], 3% sulfuric acid solution in [61], or 5% sulfuric acid solution in [26]. If only the mass loss data were compared, the corrosion rate in this study was similar to the mass loss of ordinary Portland cement concrete (OPCC) in a 3% sulfuric acid solution, as shown in Figure S13b. Moreover, the mass loss in seawater was much higher than in river water in literature [25] because of high concentrations of sulfate ions and MIC-related bacterial strains. Figure S13c shows the

comparison of corrosion rate based on strength reduction alone. The strength reduction in this study was similar to the strength reduction of OPCC in a 1.5% sulfuric acid solution. Overall, submerging in seawater was like always submerging in 1.5%-5% sulfuric acid solution based on comparison results. Generally, 1%-5% sulfuric acid solution is often used to accelerate corrosion to study the durability of concrete. Therefore, we should pay more attention to the MIC on concrete resulting from seawater.

Section S16. The influence of bacteria on concrete corrosion

Table S10. Comparison of microbially induced corrosion and chemical corrosion

	Sample type	W/C	Mass loss (%)	Strength reduction (%)	Corrosion	Immersed solution	Reference	Note
1	SSC	0.38	1.90	2.23	MIC	Submerged in seawater	Current study	Mass loss ratio: MIC/CC=2.71 or 2.40 Strength reduction ratio: MIC/CC=3.99 or 3.00
			0.70	0.56	CC			
			2.40	3.35	MIC	Tidal in seawater		
			1.00	1.12	CC			
2	OPCC	0.38	4.65		MIC	Flowing river water	[25]	Mass loss ratio: MIC/CC=1.31 or 1.36
			3.54		CC			
			4.04		MIC	Static river water		
			2.96		CC			
3	CACC	0.36	13.1		MIC	10±5 ppm H_2S(g) and a total of 10^13 cells of strains (Thiobacillus thiooxidans, T. intermedius, T. novellus, T.neapolitanus)	[81]	For OPCC, SRPC, and BFC, Mass loss ratio: MIC/AC=1.13-1.70
	OPCC		25.1					
	SRPC		27.3					
	BFC		25.4					
	CACC		13.1		AC	pH2.0 sulfuric acid solution		
	OPCC		22.2					
	SRPC		18.0					
	BFC		14.9					
4	OPCC	0.34		103.5	AC	pH2.0 sulfuric acid solution	[71]	Strength reduction ratio: MIC/CC=3.33
				344.72	AC+bacteria	pH2.0 sulfuric acid solution and A. thiooxidans	[76]	

Note: Calcium aluminate cement concrete (CACC), sulfate resistant Portland cement (SRPC), blast furnace cement (BFC). acid corrosion (AC).

Section S17. Inhibition effects of different inhibition strategies on concrete corrosion

Table S8. The corrosion inhibition effects of various methods

	Sample type	W/C	Mass loss (%)	Strength reduction (%)	Corrosion	Experimental solution	Reference	Note
1	SSC	0.38	1.90	2.23	MIC	Submerged in seawater	Current study	Mass loss ratio: BCCI/MIC=0.54 or 0.42 Strength reduction ratio: BCCI/MIC=0.50 or 0.33
			1.03	1.12	BCCI			
			2.40	3.35	MIC	Tidal in seawater		
			1.00	1.12	BCCI			
2	OPCC	0.32	4.00	30.00	AC	3% sulfuric acid solution	[82]	Mass loss ratio: SACC/OPCC=3.75 Strength reduction ratio: SACC/OPCC=0.75
	SACC		15.00	22.50				
3	OPCC	0.34	20.00	38.00	AC	pH1.1 sulfuric acid solution	[71]	Mass loss ratio: AAC/OPCC=0.43-0.65 Strength reduction ratio: AAC/OPCC=0.22-0.39. The AAC was prepared with FA, slag, river sand, and ordinary stone. The preconfigured NaOH solution was added during stirring.
	AAC		13.00	15.00				
	OPCC		7.00	23.00		pH1.6 sulfuric acid solution		
	AAC		3.00	9.00				
	OPCC		4.00	18.00		pH2.0 sulfuric acid solution		
	AAC		2.00	4.00				
4	CAC	0.36	13.1		MIC	10±5 ppm H_2S(g) and a total of 10^13 cells of strains (Thiobacillus thiooxidans, T. intermedius, T. novellus, T.neapolitanus)	[81]	Mass loss ratio: CAC/OPCC=0.52
	OPCC		25.1					
5	OPCC	0.45		2.94	CC	Submerged in artificial seawater solution	[53]	Strength reduction ratio: SCC/OPCC=1.81, or 0.78, or 0.73
				38.24		Tidal in artificial seawater solution		
				53.10		Splash in artificial seawater solution		
	SCC			5.35		Submerged in artificial seawater solution		
				29.87		Tidal in artificial seawater solution		
				38.85		Splash in artificial seawater solution		

6	OPCM	0.45	21.57	CC	Submerged in artificial seawater solution	53	Strength reduction ratio: SCM/OPCM=0.89, or 0.98, or 0.88
			27.16		Tidal in artificial seawater solution		
			48.98		Splash in artificial seawater solution		
	SCM		19.24		Submerged in artificial seawater solution		
			26.84		Tidal in artificial seawater solution		
			43.04		Splash in artificial seawater solution		
7	OPCC	0.45	11.54	CC	Artificial seawater solution (30 days)	52	Strength reduction ratio: BM/OPCC=0.20-1.21
			19.23		Artificial seawater solution (90 days)		
			32.69		Artificial seawater solution (180 days)		
	BM with GGBS and RHA		2.56-8.33		Artificial seawater solution (30 days)		
			5.13-23.33		Artificial seawater solution (90 days)		
			6.41-26.67		Artificial seawater solution (180 days)		
8	OPCC	0.38	1.82	MIC	Submerged in Persian Gulf seawater	56	Strength reduction ratio: OPCC with SF and FA/OPCC=0.47-1.12, or 0.19-1.93
			5.45		Tidal in Persian Gulf seawater		
	OPCC with SF and FA		0.85-2.04		Submerged in Persian Gulf seawater		
			1.02-5.08		Tidal in Persian Gulf seawater		

Note: Sulphoaluminate cement concrete (SACC), Alkali-activated concrete (AAC), calcium aluminate concrete (CAC), self-consolidating concrete (SCC), ordinary Portland cement mortar (OPCM), self-consolidating mortar (SCM), blended mortar (BM), ground granulated blast furnace slag (GGBS), rice husk ash (RHA), silica fume (SF), fly ash (FA), acid corrosion (AC).

References

(1) Knorst, M. T.; Neubert, R.; Wohlrab, W. Analytical methods for measuring urea in pharmaceutical formulations. *Journal of Pharmaceutical and Biomedical Analysis*. **1997**, 15(11), 1627-1632.

(2) Whiffin, V. S.; Van Paassen, L. A.; Harkes, M. P. Microbial carbonate precipitation as a soil improvement technique. *Geomicrobiology Journal*. **2007**, 24(5), 417-423.

(3) Yang, Y.; Chu, J.; Cao, B.; Liu, H.; Cheng, L. Biocementation of soil using non-sterile enriched urease-producing bacteria from activated sludge. *Journal of Cleaner Production*. **2020**, 262, 121315.

(4) Graddy, C. M.; Gomez, M. G.; DeJong, J. T.; Nelson, D. C. Native bacterial community convergence in augmented and stimulated ureolytic MICP biocementation. *Environmental Science & Technology*. **2021**, 55(15), 10784-10793.

(5) Van Paassen, L. A. Biogrout, ground improvement by microbial induced carbonate precipitation, in Biotechnology. **2009**, *Delft University of Technology*: Netherlands.

(6) Al Qabany, A.; Soga, K.; Santamarina, C. Factors affecting efficiency of microbially induced calcite precipitation. *Journal of Geotechnical and Geoenvironmental Engineering*. **2012**, 138(8), 992-1001.

(7) Martinez, B.; DeJong, J.; Ginn, T.; Montoya, B.; Barkouki, T.; Hunt, C.; Tanyu, B.; Major, D. Experimental optimization of microbial-induced carbonate precipitation for soil improvement. *Journal of Geotechnical and Geoenvironmental Engineering*. **2013**, 139(4), 587-598.

(8) De Muynck, W.; Verbeken, K.; De Belie, N.; Verstraete, W. Influence of temperature on the effectiveness of a biogenic carbonate surface treatment for limestone conservation. *Applied Microbiology and Biotechnology*. **2013**, 97(3), 1335-1347.

(9) Guo, N.; Wang, Y.; Hui, X.; Zhao, Q.; Zeng, Z.; Pan, S.; Guo, Z.; Yin, Y.; Liu, T. Marine bacteria inhibit corrosion of steel via synergistic biomineralization. *Journal of Materials Science & Technology*. **2021**, 66, 82-90.

(10) Liu, T.; Guo, Z.; Zeng, Z.; Guo, N.; Lei, Y.; Liu, T.; Sun, S.; Chang, X.; Yin, Y.; Wang, X. Marine Bacteria Provide Lasting Anticorrosion Activity for Steel via Biofilm-Induced Mineralization. *ACS Applied Materials & Interfaces*. **2018**, 10(46), 40317-40327.

(11) Zhang, L. M.; Yan, M. C.; Zhang, S. D.; Zhu, L. Y.; Umoh, A. J.; Ma, A. L.; Zheng, Y. G.; Wang, J. Q. Significantly enhanced resistance to SRB corrosion via Fe-based amorphous coating designed with high dose corrosion-resistant and antibacterial elements. *Corrosion Science*. **2020**, 164.

(12) Tang, X.; Chan, K. L.; Farzana, S.; Wai, O. W.; Leu, S. Y. Strategic planting for watershed restoration in coastal urban environment–Toward carbon sequestration by stormwater improvement. *Journal of Cleaner Production*. **2021**, 295, 126116.

(13) Mao, Q.; Shi, P.; Yin, K.; Gan, J.; Qi, Y. Tides and tidal currents in the Pearl River Estuary. *Continental Shelf Research*. **2004**, 24(16), 1797-1808.

(14) Larson, M.; Bellanca, R.; Jönsson, L.; Chen, C.; Shi, P. A model of the 3D circulation, salinity distribution, and transport pattern in the Pearl River Estuary, China. *Journal of Coastal Research*. **2005**, 21(5), 896-908.

(15) Ji, R. Y.; Xu, Q.; Jia, L. W.; Mo, S. P. Effects on the Hydrodynamics Caused by Artificial Islands of the Hong Kong-Zhuhai-Macao Bridge. in Applied Mechanics and Materials.

2012, *Trans Tech Publ*, 2085-2090.
(16) Wu, L.; Hu, C.; Liu, W. V. The sustainability of concrete in sewer tunnel-A narrative review of acid corrosion in the city of Edmonton, Canada. *Sustainability*. **2018**, 10(2), 517.
(17) Rajala, P.; Cheng, D. Q.; Rice, S. A.; Lauro, F. M. Sulfate-dependant microbially induced corrosion of mild steel in the deep sea: a 10-year microbiome study. *Microbiome*. **2022**, 10(1), 4.
(18) Tsai, Y. P. Impact of flow velocity on the dynamic behaviour of biofilm bacteria. *Biofouling*. **2005**, 21(5-6), 267-277.
(19) Committee, A. Building code requirements for structural concrete (ACI 318-05) and commentary (ACI 318R-05). **2005**, *American Concrete Institute*
(20) Hadigheh, S. A.; Gravina, R.; Smith, S. T. Effect of acid attack on FRP-to-concrete bonded interfaces. *Construction and Building Materials*. **2017**, 152, 285-303.
(21) Erbektas, A. R.; Isgor, O. B.; Weiss, W. J. An accelerated testing protocol for assessing microbially induced concrete deterioration during the bacterial attachment phase. *Cement and Concrete Composites*. **2019**, 104, 103339.
(22) Wan, M.; Li, Y.; Wang, L.; Zhang, W.; Zhang, H.; Niu, L. Insights into microbial actions on hydraulic concrete structures: Effects of concrete alkalinity on bacterial community composition and functional expression. *Construction and Building Materials*. **2021**, 280.
(23) Jiang, G.; Sun, X.; Keller, J.; Bond, P. L. Identification of controlling factors for the initiation of corrosion of fresh concrete sewers. *Water Research*. **2015**, 80, 30-40.
(24) Sun, X.; Miao, L.; Tong, T.; Wang, C. Improvement of microbial-induced calcium carbonate precipitation technology for sand solidification. *Journal of Materials in Civil Engineering*. **2018**, 30(11), 04018301.
(25) Li, Y.; Wan, M.; Du, J.; Lin, L.; Cai, W.; Wang, L. Microbial enhanced corrosion of hydraulic concrete structures under hydrodynamic conditions: Microbial community composition and functional prediction. *Construction and Building Materials*. **2020**, 248, 118609.
(26) Koushkbaghi, M.; Kazemi, M. J.; Mosavi, H.; Mohseni, E. Acid resistance and durability properties of steel fiber-reinforced concrete incorporating rice husk ash and recycled aggregate. *Construction and Building Materials*. **2019**, 202, 266-275.
(27) Qin, S.; Zou, D.; Liu, T.; Jivkov, A. A chemo-transport-damage model for concrete under external sulfate attack. *Cement and Concrete Research*. **2020**, 132, 106048.
(28) Sun, X.; Miao, L.; Wu, L.; Wang, H. Theoretical quantification for cracks repair based on microbially induced carbonate precipitation (MICP) method. *Cement and Concrete Composites*. **2021**, 118, 103950.
(29) Zou, D.; Qin, S.; Liu, T.; Jivkov, A. Experimental and numerical study of the effects of solution concentration and temperature on concrete under external sulfate attack. *Cement and Concrete Research*. **2021**, 139, 106284.
(30) Xie, J.; Jin, L.; Wu, D.; Pruden, A.; Li, X. Inhalable Antibiotic Resistome from Wastewater Treatment Plants to Urban Areas: Bacterial Hosts, Dissemination Risks, and Source Contributions. *Environmental Science & Technology*. **2022**.
(31) Nadkarni, M. A.; Martin, F. E.; Jacques, N. A.; Hunter, N. Determination of bacterial load

by real-time PCR using a broad-range (universal) probe and primers set. *Microbiology*. **2002**, 148(1), 257-266.
(32) Geets, J.; Borremans, B.; Diels, L.; Springael, D.; Vangronsveld, J.; van der Lelie, D.; Vanbroekhoven, K. DsrB gene-based DGGE for community and diversity surveys of sulfate-reducing bacteria. *Journal of Microbiological methods*. **2006**, 66(2), 194-205.
(33) Rajitha, K.; Nancharaiah, Y.; Venugopalan, V. Insight into bacterial biofilm-barnacle larvae interactions for environmentally benign antifouling strategies. *International Biodeterioration & Biodegradation*. **2020**, 149, 104937.
(34) Gao, S.-H.; Ho, J. Y.; Fan, L.; Richardson, D. J.; Yuan, Z.; Bond, P. L. Antimicrobial effects of free nitrous acid on Desulfovibrio vulgaris: implications for sulfide-induced corrosion of concrete. *Applied and Environmental Microbiology*. **2016**, 82(18), 5563-5575.
(35) Cheng, L.; House, M. W.; Weiss, W. J.; Banks, M. K. Monitoring sulfide-oxidizing biofilm activity on cement surfaces using non-invasive self-referencing microsensors. *Water Research*. **2016**, 89, 321-329.
(36) Zhong, H.; Shi, Z.; Jiang, G.; Yuan, Z. Decreasing microbially influenced metal corrosion using free nitrous acid in a simulated water injection system. *Water Research*. **2020**, 172, 115470.
(37) Vincke, E.; Verstichel, S.; Monteny, J.; Verstraete, W. A new test procedure for biogenic sulfuric acid corrosion of concrete. *Biodegradation*. **1999**, 10(6), 421-428.
(38) Wei, S.; Sanchez, M.; Trejo, D.; Gillis, C. Microbial mediated deterioration of reinforced concrete structures. *International Biodeterioration & Biodegradation*. **2010**, 64(8), 748-754.
(39) Sierra-Beltran, M. G.; Jonkers, H. M.; Schlangen, E. Characterization of sustainable bio-based mortar for concrete repair. *Construction and Building materials*. **2014**, 67, 344-352.
(40) Achal, V.; Mukherjee, A. A review of microbial precipitation for sustainable construction. *Construction and Building Materials*. **2015**, 93, 1224-1235.
(41) Luhar, S.; Gourav, S. A review paper on self healing concrete. *Journal of Civil Engineering Research*. **2015**, 5(3), 53-58.
(42) Grengg, C.; Mittermayr, F.; Koraimann, G.; Konrad, F.; Szabó, M.; Demeny, A.; Dietzel, M. The decisive role of acidophilic bacteria in concrete sewer networks: A new model for fast progressing microbial concrete corrosion. *Cement and Concrete Research*. **2017**, 101, 93-101.
(43) Ouyang, X.; Koleva, D.; Ye, G.; Van Breugel, K. Understanding the adhesion mechanisms between CSH and fillers. *Cement and Concrete Research*. **2017**, 100, 275-283.
(44) Cheng, S.; Wu, Z.; Wu, Q.; Chen, X.; Shui, Z.; Lu, J. X. Degradation characteristics of Portland cement mortar incorporating supplementary cementitious materials under multi-ions attacks and drying-wetting cycles. *Journal of Cleaner Production*. **2022**, 132378.
(45) Cheng, S.; Shui, Z.; Gao, X.; Yu, R.; Sun, T.; Guo, C.; Huang, Y. Degradation mechanisms of Portland cement mortar under seawater attack and drying-wetting cycles. *Construction and Building Materials*. **2020**, 230, 116934.
(46) Jiang, G.; Zhou, M.; Chiu, T. H.; Sun, X.; Keller, J.; Bond, P. L. Wastewater-Enhanced

Microbial Corrosion of Concrete Sewers. *Environmental Science Technology*. **2016**, 50(15), 8084-92.

(47) Sheoran, A.; Sheoran, V.; Choudhary, R. Bioremediation of acid-rock drainage by sulphate-reducing prokaryotes: a review. *Minerals Engineering*. **2010**, 23(14), 1073-1100.

(48) Rosnes, J. T.; Torsvik, T.; Lien, T. Spore-forming thermophilic sulfate-reducing bacteria isolated from North Sea oil field waters. *Applied and Environmental Microbiology*. **1991**, 57(8), 2302-2307.

(49) Thauer, R. K.; Stackebrandt, E.; Hamilton, W. A. Energy metabolism and phylogenetic diversity of sulphate-reducing bacteria. Sulphate-reducing Bacteria. **2007**, *Cambridge University Press*, 1-38.

(50) Wu, M.; Wang, T.; Wu, K.; Kan, L. Microbiologically induced corrosion of concrete in sewer structures: A review of the mechanisms and phenomena. *Construction and Building Materials*. **2020**, 239, 117813.

(51) Kayombo, S.; Mbwette, T.; Mayo, A. W.; Katima, J.; Jorgensen, S. Modelling diurnal variation of dissolved oxygen in waste stabilization ponds. *Ecological Modelling*. **2000**, 127(1), 21-31.

(52) Srinivas, D.; Ramagiri, K. K.; Kar, A.; Adak, D.; Farsangi, E. N.; Dutta, S. Experimental characterization of quaternary blended mortar exposed to marine environment using mechanical strength, corrosion resistance and chemical composition. *Journal of Building Engineering*. **2021**, 42, 102822.

(53) Alaghebandian, N.; Mirvalad, S.; Javid, A. A. S. Durability of self-consolidating concrete and mortar mixtures containing ternary and quaternary cement blends exposed to simulated marine environment. *Construction and Building Materials*. **2020**, 259, 119767.

(54) Rashad, A. M.; Ouda, A. S. Effect of tidal zone and seawater attack on high-volume fly ash pastes enhanced with metakaolin and quartz powder in the marine environment. *Microporous and Mesoporous Materials*. **2021**, 324, 111261.

(55) Hewayde, E.; Nehdi, M.; Allouche, E.; Nakhla, G. Effect of mixture design parameters and wetting-drying cycles on resistance of concrete to sulfuric acid attack. *Journal of Materials in Civil Engineering*. **2007**, 19(2), 155-163.

(56) Hassanabad, H. T.-R. M. A. G. M. A. M. G. Comparing the durability of self-compacting concrete containing silica fume and fly ash with conventional concrete in the marine environment. *Journal of Xi'an University of Architecture & Technology*. **2021**(2), 335-356.

(57) Rashad, A. M.; Sadek, D. M. An exploratory study on alkali-activated slag blended with microsize metakaolin particles under the effect of seawater attack and tidal zone. *Arabian Journal for Science and Engineering*. **2022**, 47(4), 4499-4510.

(58) Cai, Y.; Xuan, D.; Hou, P.; Shi, J.; Poon, C. S. Effect of seawater as mixing water on the hydration behaviour of tricalcium aluminate. *Cement and Concrete Research*. **2021**, 149, 106565.

(59) Sun, Y.; Zhang, Y.; Cai, Y.; Lam, W. L.; Lu, J. X.; Shen, P.; Poon, C. S. Mechanisms on accelerating hydration of alite mixed with inorganic salts in seawater and characteristics of hydration products. *ACS Sustainable Chemistry & Engineering*. **2021**, 9(31), 10479-

10490.
(60) Ismail, I.; Bernal, S. A.; Provis, J. L.; Hamdan, S.; van Deventer, J. S. Microstructural changes in alkali activated fly ash/slag geopolymers with sulfate exposure. *Materials and Structures*. **2013**, 46, 361-373.
(61) Gu, T.; Jia, R.; Unsal, T.; Xu, D. Toward a better understanding of microbiologically influenced corrosion caused by sulfate reducing bacteria. *Journal of Materials Science & Technology*. **2019**, 35(4), 631-636.
(62) Barak-Gavish, N.; Frada, M. J.; Ku, C.; Lee, P. A.; DiTullio, G. R.; Malitsky, S.; Aharoni, A.; Green, S. J.; Rotkopf, R.; Kartvelishvily, E. Bacterial virulence against an oceanic bloom-forming phytoplankter is mediated by algal DMSP. *Science Advances*. **2018**, 4(10), eaau5716.
(63) De Carvalho, C. C. Marine biofilms: a successful microbial strategy with economic implications. *Frontiers in Marine Science*. **2018**, 5, 126.
(64) Ling, A. L.; Robertson, C. E.; Harris, J. K.; Frank, D. N.; Kotter, C. V.; Stevens, M. J.; Pace, N. R.; Hernandez, M. T. Carbon dioxide and hydrogen sulfide associations with regional bacterial diversity patterns in microbially induced concrete corrosion. *Environmental Science & Technology*. **2014**, 48(13), 7357-7364.
(65) Cayford, B. I.; Dennis, P. G.; Keller, J.; Tyson, G. W.; Bond, P. L. High-throughput amplicon sequencing reveals distinct communities within a corroding concrete sewer system. *Applied and Environmental Microbiology*. **2012**, 78(19), 7160-7162.
(66) Yongsiri, C.; Vollertsen, J.; Rasmussen, M.; Hvitved-Jacobsen, T. Air-water transfer of hydrogen sulfide: an approach for application in sewer networks. *Water Environment Research*. **2004**, 76(1), 81-88.
(67) Barridge, J. K.; Shively, J. Phospholipids of the Thiobacilli. *Journal of Bacteriology*. **1968**, 95(6), 2182-2185.
(68) Kelly, D. P. Stable sulfur isotope fractionation and discrimination between the sulfur atoms of thiosulfate during oxidation by Halothiobacillus neapolitanus. *FEMS Microbiology Letters*. **2008**, 282(2), 299-306.
(69) Ariffin, M.; Bhutta, M.; Hussin, M.; Tahir, M. M.; Aziah, N. Sulfuric acid resistance of blended ash geopolymer concrete. *Construction and Building Materials*. **2013**, 43, 80-86.
(70) Kabus, O.; Kolomiiets, Y.; Lykhohrai, V. Estimation of corrosion resistance of modifying concrete in a solution of sulfuric acid. in MATEC Web of Conferences. **2018**, *EDP Sciences*, 03006.
(71) Xie, Y.; Lin, X.; Ji, T.; Liang, Y.; Pan, W. Comparison of corrosion resistance mechanism between ordinary Portland concrete and alkali-activated concrete subjected to biogenic sulfuric acid attack. *Construction and Building Materials*. **2019**, 228, 117071.
(72) Siad, H.; Lachemi, M.; Sahmaran, M.; Hossain, K. M. A. Effect of glass powder on sulfuric acid resistance of cementitious materials. *Construction and Building Materials*. **2016**, 113, 163-173.
(73) Israel, D.; Macphee, D. E.; Lachowski, E. Acid attack on pore-reduced cements. *Journal of Materials Science*. **1997**, 32(15), 4109-4116.
(74) Huber, B.; Hilbig, H.; Drewes, J. E.; Müller, E. Evaluation of concrete corrosion after short-and long-term exposure to chemically and microbially generated sulfuric acid.

Cement and Concrete Research. **2017**, 94, 36-48.
(75) Monteny, J.; Vincke, E.; Beeldens, A.; De Belie, N.; Taerwe, L.; Van Gemert, D.; Verstraete, W. Chemical, microbiological, and in situ test methods for biogenic sulfuric acid corrosion of concrete. *Cement and Concrete Research*. **2000**, 30(4), 623-634.
(76) Yousefi, A.; Allahverdi, A.; Hejazi, P. Accelerated biodegradation of cured cement paste by Thiobacillus species under simulation condition. *International Biodeterioration & Biodegradation*. **2014**, 86, 317-326.
(77) Sand, W.; Bock, E. Concrete corrosion in the Hamburg sewer system. *Environmental Technology*. **1984**, 5(12), 517-528.
(78) Okabe, S.; Odagiri, M.; Ito, T.; Satoh, H. Succession of sulfur-oxidizing bacteria in the microbial community on corroding concrete in sewer systems. *Applied and Environmental Microbiology*. **2007**, 73(3), 971-980.
(79) Chalee, W.; Cheewaket, T.; Jaturapitakkul, C. Enhanced durability of concrete with palm oil fuel ash in a marine environment. *Journal of Materials Research and Technology*. **2021**, 13, 128-137.
(80) Khan, M. S.; Yang, C.; Zhao, Y.; Pan, H.; Zhao, J.; Shahzad, M. B.; Kolawole, S. K.; Ullah, I.; Yang, K. An induced corrosion inhibition of X80 steel by using marine bacterium Marinobacter salsuginis. *Colloids and Surfaces B: Biointerfaces*. **2020**, 189, 110858.
(81) Ehrich, S.; Helard, L.; Letourneux, R.; Willocq, J.; Bock, E. Biogenic and chemical sulfuric acid corrosion of mortars. *Journal of Materials in Civil Engineering*. **1999**, 11(4), 340-344.
(82) Yang, Y.; Ji, T.; Lin, X.; Chen, C.; Yang, Z. Biogenic sulfuric acid corrosion resistance of new artificial reef concrete. *Construction and Building Materials*. **2018**, 158, 33-41.
